# Whole genome experimental maps of DNA G-quadruplexes in multiple species

**DOI:** 10.1093/nar/gkz179

**Published:** 2019-03-20

**Authors:** Giovanni Marsico, Vicki S Chambers, Aleksandr B Sahakyan, Patrick McCauley, Jonathan M Boutell, Marco Di Antonio, Shankar Balasubramanian

**Affiliations:** 1 Cancer Research UK, Cambridge Research Institute, Li Ka Shing Centre, Cambridge CB2 0RE, UK; 2 Department of Chemistry, University of Cambridge, Cambridge CB2 1EW, UK; 3 Illumina Cambridge Ltd., Chesterford Research Park, Little Chesterford, Saffron Walden, Essex CB10 1XL, UK; 4 School of Clinical Medicine, University of Cambridge, Cambridge CB2 0SP, UK

## Abstract

Genomic maps of DNA G-quadruplexes (G4s) can help elucidate the roles that these secondary structures play in various organisms. Herein, we employ an improved version of a G-quadruplex sequencing method (G4-seq) to generate whole genome G4 maps for 12 species that include widely studied model organisms and also pathogens of clinical relevance. We identify G4 structures that form under physiological K^+^ conditions and also G4s that are stabilized by the G4-targeting small molecule pyridostatin (PDS). We discuss the various structural features of the experimentally observed G-quadruplexes (OQs), highlighting differences in their prevalence and enrichment across species. Our study describes diversity in sequence composition and genomic location for the OQs in the different species and reveals that the enrichment of OQs in gene promoters is particular to mammals such as *mouse* and *human*, among the species studied. The multi-species maps have been made publicly available as a resource to the research community. The maps can serve as blueprints for biological experiments in those model organisms, where G4 structures may play a role.

## INTRODUCTION

G-quadruplexes (G4s) are non-canonical structures that can arise in single-stranded guanine-rich DNA and RNA sequences (1–3). They form through Hoogsteen hydrogen-bonding of four guanines, to form a planar G-tetrad (1). The stacking of two or more of these G-tetrads defines the four-stranded G4, a knot-like structure with high thermodynamic stability under physiological conditions (3,4). G4 structures are stabilized by monovalent cations, with stabilization strength according to the following order: K^+^> Na^+^> NH_4_^+^>> Li^+^ (5–7). DNA G4s have been visualised in *human* cells (8), and they have been implicated in various biological processes, such as transcription, DNA replication, DNA damage and telomere maintenance (9–11).

Several methods have been devised in the last decade to study the formation and stability of these structures *in vitro* (12,13) or to computationally predict their formation in genomic contexts (14–18). Biophysical studies have shed light on factors that influence G4 formation but are typically low throughput and limited to sequences of short length. Conversely, computational predictions can be applied to any given genome but lack a thorough experimental validation, and rather employ algorithms derived from experimental data on a small number of sequences. Folded G4s have been detected in small genomes by polymerase pausing using PacBio SMRT sequencing (19), though this approach has not yet been scaled to larger genomes. To overcome all such limitations and also go beyond computational prediction, we recently developed G4-seq to experimentally detect and map G4 structures in a way that is scalable to the *human* genome (20). This method identified hundreds of thousands (*n* = 716310) of G4 forming structures and has revealed important features that govern G4 formation and stability including the relevance of genomic sequence context. The *human* G4 map was generated using purified DNA and has served as a reference to interpret biological studies (21–24). The initial G4-seq *human* genome dataset revealed some shortfalls in the various G4 computational prediction algorithms, which can lead to an over- or under-estimation of G4 structures. Furthermore, the dataset (20) was largely made up of non-canonical G4s with long loops, bulges or comprising only two G-tetrads, all of which are difficult to predict with accuracy from the primary DNA sequence alone. Some improvements in sequence-based G4 prediction have been recently made via computational approaches that have employed the G4-seq dataset for training a machine learning model (17,25,26).

**Figure 1.**
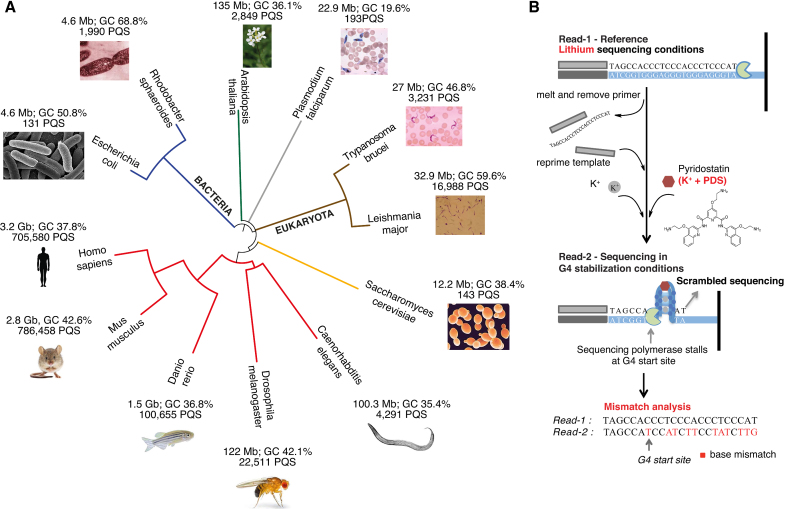
The 12 species in this study and details of the improved G4-seq method. (**A**) Phylogenetic representation of the 12 species analysed, with details on genome size, GC richness of the analysed chromosomes and the count of PQS motif of the form G_3+_L_1–12_ (see Materials and Methods). (**B**) The improved G4-seq method, with highlighted in red the different steps compared to the published G4-seq method: first sequencing run (Read-1) under Li^+^ instead of Na^+^ conditions; maximal stabilization achieved using PDS, a selective and potent G4 stabilising ligand, performed as K^+^+PDS.

We applied the refined G4-seq protocol to the genomes of 12 different species, comprising important model organisms and pathogens of clinical relevance (see list in Supplementary Table S1 and Figure 1A). As part of this study, we also addressed some limitations of the original G4-seq approach (20), which included poor coverage at high GC-rich regions, limiting its use for G-rich genomes or regions, and insufficient spatial resolution to disentangle G4s in close proximity (Figure 1B). The comparative G4 maps generated provide important insights into G4 structural classes across species, the key sequence features that determine different patterns of G4 formation and the relevance of G4 localization across genomes.

## MATERIALS AND METHODS

### Species naming convention

The full scientific name of each of the 12 species analysed in this study are reported at Supplementary Table S1, together with the short name used as convention throughout the text in this work. The short name used is either a concise version of the full scientific name (e.g. Drosophila instead of *Drosophila melanogaster*, or C. elegans instead of *Caenorhabditis elegans*) or the common name used in the field for that species (e.g. human, mouse and zebrafish for *Homo sapiens, Mus Musculus* and *Danio rerio* respectively), as detailed in Supplementary Table S1.

### PQS motifs

PQS (Putative G-Quadruplex Sequences) are computationally defined sequence motifs that have features compatible with G-quadruplex formation. A PQS generally consist of stretches at least four G runs (i.e. two or more consecutive Gs) separated by nucleotide stretches of different length (loops). The PQS used in this study comply to the following regular expressions:

1. **G_3+_L_1–7_** = canonical PQS, with at least three tetrads and loops of length up to seven nucleotides: ‘([gG]{3,}\w{1,7}){3,}[gG]{3,}’;
2. **G_3+_L_1–12_** = extended canonical PQS, with at least three tetrads and longer loops up to 12 nucleotides: ‘([gG]{3,}\w{1,12}){3,}[gG]{3,}’;
3. **G_2_L_1–12_** = two-tetrads PQS, with loops up to 12 nucleotides: ‘([gG]{2}\w{1,12}){3,}[gG]{2}’;
4. **G_3+_L_8–12_** = extended canonical PQS only with longer loops, i.e., with at least one loop of length between 8 and 12 nucleotides. Those are sequences from G_3+_L_1–12_ not including G_3+_L_1–7_;
5. **G_2+_L_1–12_** = motif comprising PQS with two or more tetrads and loops up to 12 nucleotides: ‘([gG]{2,}\w{1,12}){3,}[gG]{2,}’.

### Preparation of sequencing libraries of different organisms

Purified genomic DNA (1–5 μg) from the following organisms was kindly provided by colleagues in the UK for use in G4-seq version 2: *Arabidopsis thaliana* (Professor David Baulcombe, Department of Plant Sciences, University of Cambridge), *Caenorhabditis elegans* (N2 strain, Dr Eric Miska, The Gurdon Institute, Cambridge), *Danio rerio* (Dr Angeleen Fleming, Department of Physiology, University of Cambridge), *Drosophila melanogaster* (Professor Steve Russell, Department of Genetics, University of Cambridge), *DT40* (Dr Julian Sale, MRC Laboratory of Molecular Biology, Cambridge), *Plasmodium falciparum* (Professor Chris Newbold, Radcliffe Department of Medicine, Oxford and Dr Matt Berriman, Sanger Institute, Cambridgeshire), *Rhodobacter sphaeroides* (Illumina, UK), and *Trypanosoma brucei* (EATR01125 strain, Dr Mark Carrington, Department of Biochemistry, Cambridge). Purified genomic DNA from *Leishmania major* was purchased from ATCC^®^ (30012D™, 2 μg) and *Escherichia coli* non-methylated DNA from Zymo Research (ER2925 strain, D5016). *Human* genomic DNA was extracted from *human* HEK-293T cells, cultured as previously described in section 7.1.6, by phenol/chloroform extraction, using a phenol:chloroform:isoamyl alcohol solution (25:24:1, Thermo Scientific). The resulting aqueous layer was purified and concentrated using ethanol precipitation at –20°C overnight, to give the purified genomic material. Genomic DNA was obtained from yeast, *Saccharomyces cerevisiae* purchased from Sigma Aldrich (Type II, YSC2). The yeast (50 mg) was hydrated overnight at 20°C, lysed using a proteinase K containing buffer (10 mM Tris pH 8.0, 100 mM NaCl, 10 mM EDTA pH 8.0, 0.5% SDS and 2 μl Proteinase K) at 56°C for 5 h and then incubated at 4°C overnight. Phenol/chloroform extraction and ethanol precipitation were then used to obtain the purified genomic material. Finally, *mouse* (*Mus musculus*) genomic DNA was extracted from skin tissues of a 12-week-old male *mouse* (C57BL/6J strain, standard JAX reference strain from Charles River), provided by Biological resources unit, CRUK-CI genomics core, using DNeasy Blood and Tissues kit (Qiagen, 69504) according to the manufacturer's protocol. The integrity of all samples was assessed using a genomic DNA screentape on the Tapestation and DNA was quantified using dsDNA HS assay kit (Qubit). Genomic DNA samples were then sonicated and the library prepared, as in (20). Only DNA from *H. sapiens* was used with TruSeq Nano DNA LT Library Prep Kit, however all genomic DNA was used with PCR-Free Library Prep Kit. Modified sequencing buffers were prepared as previously described (20), with the only difference being the addition of the small-molecule PDS to the K^+^ instead of the Na^+^ buffer.

### Raw processing: alignment and mismatch calculation

Fastq files are generated through a customized protocol, where DNA fragments are sequenced two times with 150 bp, similarly to a paired-end protocol. However, the fragment read is not ‘flipped’ at Read-2 but just re-sequenced in different buffer conditions, as detailed in Chambers *et al.* (20). Fasta genome files were downloaded from public repositories for each species (Supplementary Table S4). The main processing steps are as follows:

1. *Fastq* files from *Read-1* are aligned to the respective reference genomes using the bwa mem aligner (http://bio-bwa.sourceforge.net).
2. Aligned *bam* files are processed with a customized script that converts *bam* to *bed* files (*bedtools bamtobed*) and then extracts the alignment with the highest mapping quality (MapQ) for each read (*bedtools groupBy*).
3. An *R* script (27) takes in input the *Read-1* and *Read-2 fastq* files and the *bed* alignment files generated at step 2 and calculates for each read the quality scores at *Read-1* and *Read-2*, the delta quality score (i.e., the quality drop *Read-1* minus *Read-2*) and the percentage of mismatches (mismatches %) between *Read-1* and *Read-2*. The *MismatchAnalyzer R* script is deposited as part of the Supplementary Code in Chambers *et al.* (20).
4. The results are saved to mismatch files.

### Mismatch combination and hit calling

Mismatch files generated as explained in the previous paragraph are processed as follows:

1. Each mismatch file is split by chromosome and each read is further split in single bases and the mismatches % is assigned to the first 50 bases belonging to a give read (*bedtools makewindows*).
2. Alignments shorter than 50 bases are filtered out.
3. Another customized *R* script (27) loads all split files of each chromosome and merges the mismatch values, i.e. the mismatch value for each base is averaged throughout all the bases in all files that map at that location. The *MismatchCombiner_2 R* script is deposited as part of the Supplementary Code in Chambers *et al.* (20).
4. The coverage for each base is stored as well during the procedure, generating two output *bedGraph* files: the mismatch and the coverage files. Coverage files are also combined and regions with no coverage are extracted (*bedtools complement*). Mismatch files are stored as part of the GEO submission (accession GSE110582) and have the prefix corresponding to the species they refer to and the suffix for the strand (forward/reverse) or the condition (K^+^/K^+^+PDS) they refer to (full explanation in Supplementary File S4). Similarly, coverage files are stored at the same location and have the string ‘.cov’ added within the file name (see naming details in Supplementary File S4).
5. The combined mismatch files are merged together, and contiguous regions with maximal percentage mismatch value above 25 for K^+^ and above 35 for K^+^+PDS are extracted and stored in *bed* files (commands *bedtools merge*, option *score = ‘max’*). Those positively scoring regions are defined as OQs, i.e. Observed G-Quadruplexes: the files have been deposited separately for forward and reverse strands as part of the GEO submission (accession GSE110582) and the naming is explained in Supplementary File S4 (see ‘OQs reverse strand’ and ‘OQs forward strand’). For instance, the file ‘GSM3003548_Mouse_all_w15_th-1_minus.hits.max.PDS.w50.35.bed.gz’ indicates OQs detected in Mouse on the forward strand in the K^+^+PDS condition.

### Characterization of the improved G4-seq method

To quantify the effect of Li^+^ sequencing and PCR-free library preparation methods, as compared to PCR-amplified, Na^+^ sequencing, we analysed in-depth the coverage at *Chr 1*, which contains over 60k PQS of the form G_3+_L_1–12_ over 250 Mb (Supplementary Data). We made sure that the subsets analysed would have similar coverage in all the compared methods: 8.2 per-nt per-strand in the published G4-seq method (label PAPER in figures); 7.4 in the Li^+^, PCR-amplified method (label PCR in figures); 8.6 for the Li^+^, PCR-free method (label PCR-FREE in figures). Coverage was inspected at PQS motifs with different loop length: 1–12, and the sub-categories 1–3, 4–7 and 8–12, and compared across sequencing methods. Coverage was also inspected at over 9 million windows of 50 nt, overlapping 25 nt with each other, covering the entire *Chr 1*. Windows exhibiting GC content >70% were calculated (*n =* 105 420) and further subset to those also containing PQS motifs (G_3+_L_1–12_, *n* = 38 329), and inspected for coverage. For the impact of averaging window size during analysis, we compared the case with 50 nt to the one with 150 nt. We re-analysed in the same way *Chr 1* of the Li^+^, PCR-free *human* library, and assessed the number of PQS motifs (G_3+_L_1–7_) present in OQs and detected for both window sizes, the average OQs region size and the number of OQs containing more than a single G_3+_L_1–7_ motif.

### Sequence analysis for G4 structural features

Hit files, also called OQs (Observed Quadruplexes), are then intersected to different predicted G-quadruplexes (PQS) files (see Table 2): G_3+_L_1–7_, G_3+_L_8–12_, G_2_L_1–12_ (see PQS motifs in Materials and Methods). The intersection of each one of these files with the OQs in the respective species is calculated, and conversely also the overlap of the OQs to each PQ file (*bedtools intersect*, by swapping the *–a* and *–b* options for the two cases). To generate the pie charts, the number of OQs in each PQS category was calculated, and OQs were assigned hierarchically to the first matching category (according to the order presented above). OQs without any coverage (not even partial coverage) were classified as non-covered; PQS not scoring and overlapping areas with no coverage were extracted (*bedtools intersect*) as well as PQS not overlapping regions with no coverage (i.e. entirely covered; *bedtools intersect* option *-v*).

### Genomic region analysis

The gene annotation files used in this analysis have been downloaded from publicly available databases, as listed in Supplementary Table S4. First, the different transcript regions, such as 5′UTR, exons, introns, lncRNA and promoter regions have been extracted from the .*gff* files. For promoters, we used as definition 1 kb upstream of the transcription start site (TSS); for TSS regions, we used ±1 kb from the TSS. Each annotation so generated has been intersected (*bedtools intersect*) with the OQ files in the respective species, and the overlap counted. Random genome-wide shuffling of the OQ file has been performed three independent times (*bedtools shuffle*), and the overlap has been assessed for the random case. The fold enrichment of the actual overlap divided by the average random overlap has been calculated for each species separately, and the standard error of the mean fold enrichment computed.

### Cross-species promoter co-occurrence analysis of OQs

The transcription start sites (TSS) of all genes in the *human* genome have been retrieved and the corresponding orthologous genes have been cross-mapped in five other species. *Human, mouse, Drosophila, C. elegans, zebrafish* and *Saccharomyces* genomes were considered for this analysis, due to the presence of well-annotated assemblies available programmatically from the Ensembl genome database (http://www.ensembl.org) and because they represent the most related species to *human* in this study. For each considered genome, the TSSs have been retrieved along with the corresponding gene names (Ensembl gene IDs for ortholog genes in species other than *human*), chromosome name and strand, transcript start and end coordinates. The retrieval was done using the *biomaRt* (28) library in *R* (27), which provides an *R* interface to the *Biomart* data query and retrieval system of Ensembl genome database. *Human* gene set was taken as a reference, with all the corresponding ortholog information pulled from the other species. The versions of the genome assemblies for which the genomic coordinates were retrieved were *hg38* (*GRCh38*, for *human*), *mm10* (*GRCm38*, for *mouse*), *dm6* (*BDGP6*, for *Drosophila*), *ce11* (*WBcel235*, for *C. elegans*), *danRer10* (*GRCz10*, for *zebrafish*) and *sacCer3* (*R64-1-1*, for *Saccharomyces*). These assemblies matched those used for all the OQs analyses, except for *hg38* and *ce11* for *human* and *C. elegans*, respectively. For the latter two species, the obtained genomic coordinates were then converted into the *hg19* (*GRCh37*) and *ce10* (*WormBaseWS220*) versions, using the program liftOver (http://genome.ucsc.edu/cgi-bin/hgLiftOver). Promoter regions were defined as 1 kb upstream of the TSS for each species, and the maximal mismatch value in the regions was calculated, and hierarchical cluster analysis on a matrix where rows represent the TSS (*n* = 24 164 *human* TSS, of which 17 400 have at least one orthologues) and column represent the mismatch values in the 6 species considered (Supplementary File S2).

## RESULTS

### Sequencing G4s in multiple genomes

Details of the previous limitations and current refinements of G4-seq and the characterization of the improved method are described in the paragraph ‘The improved G4-seq method’ of the Supplementary Data. In essence, the use of Li^+^ in the initial sequencing run improved the fraction of PQS sites with sequencing coverage, as compared to using Na^+^, especially for G4 motifs with loops shorter than four nucleotides. The use of a PCR-free protocol eliminated certain biases and improved coverage in regions with GC-content over 70%, including those containing PQS motifs.

We applied the revised G4-seq method to the genomic DNA of 12 different species that were chosen for being either important model organisms such as *mouse, Drosophila* and *C. elegans*, or important pathogens, such as *Leishmania* and *E. coli* (Table 1; see Supplementary Table S1 for genome sources). The chosen genomes also provided natural variation in genome size, GC content and the number/density of computationally predicted G4s (PQS motifs) (Table 1 and Supplementary Figure S1) enabling a comparative assessment of G4 formation in various genomic contexts. Details about the sequencing yield and the total number of different motifs of PQS used in the following analyses are listed in Table 1 and Supplementary Table S2 (Materials and Methods).

**Table 1.** Genome size, GC and PQS content for the 12 species. # PQS G_3+_L_1–12_ = number of motifs of the form G_3+_L_1–12_ in the genome; density of PQS = 1000 × PQS_total_size / (2 x genome_size); depth is calculated per each strand separately.

Genome	Genome size (Mb)	GC content (%)	# PQS G_3+_L_1–12_	PQS G_3+_L_1–12_ density	Seq depth K^+^ (single nt coverage)	Seq depth PDS (single nt coverage)
*human*	3095.69	37.8%	705 580	4.17	12.7	12.4
*mouse*	2730.87	42.6%	786 458	5.94	9.8	5.1
*zebrafish*	1371.72	36.8%	100 655	1.19	25.7	5.7
*Drosophila*	143.73	42.1%	22 511	2.96	12.2	6.4
*C. elegans*	100.29	35.4%	4291	0.89	49.8	49.6
*Saccharomyces*	12.16	38.4%	143	0.25	122.3	144.5
*Leishmania*	32.86	59.6%	16 988	8.21	14.8	20.3
*Trypanosoma*	35.83	46.8%	3231	1.52	9.5	10.1
*Plasmodium*	23.33	19.6%	193	0.73	45.4	22.9
*Arabidopsis*	119.67	36.1%	2849	0.46	20.1	10.2
*E. coli*	4.64	50.8%	131	0.42	46.1	52.4
*Rhodobacter*	4.60	68.8%	1990	7.57	21.4	577.9

The key difference in the analysis, compared to the previous approach (20), is the windows size and scoring thresholds. Sequences in *Read-1* (in Li^+^), where G4 formation is less favoured, are compared to the same sequences acquired in *Read-2* (in either K^+^ or K^+^+PDS) where the G4 formation is favoured. The mismatches are calculated and averaged for each genomic location with windows of 50 bp (see Methods for details). Thresholds to identify observed G-quadruplex (OQ) have been set to 25% for K^+^ and 35% for K^+^+PDS after inspecting the mismatch distribution for PQS motifs, detailed in the next section. Mismatch percentage, coverage and OQs can be visualized as tracks in a genome browser such as IGV (29). Representative tracks are shown in Supplementary Figure S2.

### G-quadruplex maps for 12 model species

**Figure 2.**
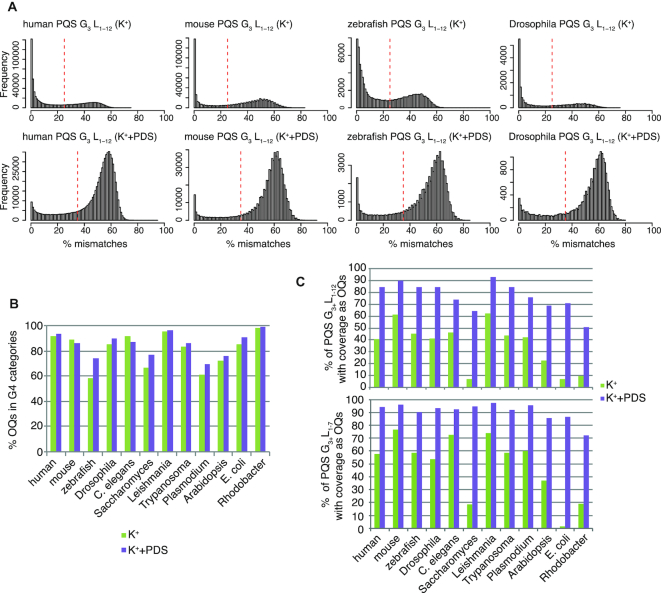
Overall features of OQs and PQS scoring. (**A**) Histograms showing the distribution of mismatch percentage levels for the G_3+_L_1–12_ PQS motifs; x-axes: mismatch percentage; y-axes: frequency, i.e. counts per bin. Top row: K^+^ condition; bottom row: PDS condition. (**B**) Bar plot indicating the method specificity, i.e. percentage of OQs assigned to the relaxed G4 category (G_2+_L_1–12_, see Materials and Methods) in K^+^ (green bars) and PDS (purple bars). (**C**) Bar plot indicating the method sensitivity, i.e. the percentage of the G_3+_L_1–12_ (top) and G_3+_L_1–7_ (bottom) PQS motifs scoring as OQs in K^+^ (green bars) and PDS (purple bars). Percentage is calculated as the number of PQS scoring divided by the number of PQS with coverage.

After calculating the mismatch percentages for all 12 species in both K^+^ and PDS conditions, we inspected the distribution of mismatches for PQS (G_3+_L_1–12_, Figure 2A and Supplementary Figure S3). Notably, the score distribution is essentially bimodal, with a consistent proportion of PQS exhibiting very low mismatches close to 0%, indicating that many predicted G4s do not form stably at physiological K^+^ concentration. However, on addition of the small-molecule stabiliser PDS, the majority of these predicted G4s show mismatches >40%. This shift is evident in the distributions for all species (Figure 2A and Supplementary Figure S3), demonstrating that insufficient G4 stability, rather than technical artefacts, determines the absence of G4 scoring under K^+^ conditions. The bimodal nature of these distributions also shows that the choice of the OQs thresholds (25% for K^+^ and 35% for K^+^+PDS) is appropriate, as the two PQS populations with high and low mismatches (stably forming and not forming, respectively) are split correctly.

We define specificity as the proportion of OQs that satisfy the minimal requirement for a G4 to have at least two tetrads (i.e. G_2+_L_1–12_). We reasoned that sequences that do not conform to this relaxed G4 motif are likely false positives (Materials and Methods). We define sensitivity as the proportion of PQS (G_3+_L_1–12_) that are identified as OQs, since there is consensus supporting *in vitro* G4 formation for G_3+_L_1–12_ motifs (30,31). We observed that the majority of OQs exhibited high specificity (>80% for most species, Figure 2B). Some species exhibited higher specificity, such as the bacterial genomes, *human, mouse, Drosophila, C. elegans* and *Leishmania*, while others gave lower specificity, such as *Plasmodium, Saccharomyces* and *zebrafish*, especially in the K^+^ condition. The use of a higher threshold in K^+^+PDS, made possible by the extra G4 stabilization provided by PDS, helps reduce false positives and other non-G4 related sequencing errors (Figure 2B). Crucially, the good specificity did not compromise the sensitivity of the assay. Figure 2C shows that under K^+^ conditions, most species show a percentage of PQS (G_3+_L_1–12_) scoring in the range 40–60% (and 55–75% for the more stable G_3+_L_1–7_ category), but there are some notable exceptions with lower scoring percentages, such as *E. coli, Rhodobacter, Arabidopsis* and *Saccharomyces*. The sensitivity increases strongly under PDS stabilizing conditions for all species (>70% for 10 out of 12 species, and >50% for the other two).

### Structural categories of OQS

**Figure 3.**
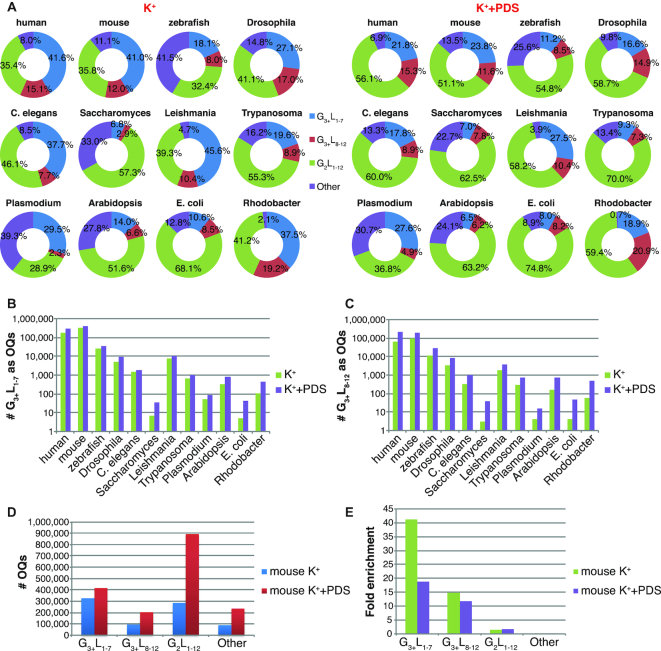
Structural categories of OQs hits. (**A**) OQs assigned to one of the PQS motifs, G_3+_L_1–7_, G_3+_L_8–12_, G_2_L_1–12_ or Other, i.e. not in any of the previous categories (Methods), in K^+^ (ring charts on the left) or PDS (ring charts on the right). The motifs counts associated with this graph are reported in Table 2 (K^+^) and Supplementary Table S3 (K^+^+PDS). (**B**) Bar plot showing the prevalence of G_3+_L_1–7_ motifs identified as OQs (logarithmic scale) for K^+^ (green bars) and PDS (purple bars). (**C**) Same as B) but shown for the G_3+_L_8–12_ motifs. (**D**) Bar plot showing the number of OQs stratified by structural categories as in A) for mouse in K^+^ (blue bars) and PDS (red bars) conditions. (**E**) Fold enrichment of the OQs structural categories in mouse, shown in D), assessed as the ratio of the actual count divided by the average (*n* = 3) occurrence measured after random permutation in the genome (Materials and Methods).

G4s can diverge from the commonly used motif comprising four runs of three guanines separated by loops up to seven nucleotides in length (14). Variants of G4s with longer loops, interruptions in the G-run such as mismatches or bulges (32), and G4s with only two G-tetrads are also possible though typically exhibit lower stability (2,33). From the OQs identified, we classified different structural categories, from the most stable to the least stable, based on previous biophysical knowledge (Methods) (20,21). The categories considered were, starting from the highest predicted stability, G_3+_L_1–7_ (standard, three-tetrad G4 motif), G_3+_L_8–12_ (longer loops, three-tetrad G4 motif), G_2_L_1–12_ (two-tetrad G4 motif), Other (sequences that cannot be directly ascribed by any of the classifier presented in this work). Note that the third category G_2_L_1–12_ includes both two-tetrad motifs and sequences with bulges, i.e. three-tetrads structures with interruptions in the G run, which cannot be unambiguously assigned without structural analysis, such as NMR spectroscopy. The relative proportions of each of the different G4 categories in K^+^ varies across species, as shown in the pie charts (Figure 3A and Table 2): the canonical PQS G_3+_L_1–7_ motif (blue sector in the pie charts), i.e. those sequences considered to have high stability, occupy a large fraction of the OQs identified in K^+^ conditions in *human, mouse, Leishmania, C. elegans* and *Rhodobacter*, while it represents only a minority for *zebrafish, Trypanosoma, Arabidopsis, E. coli* and *Saccharomyces*. Longer loop PQS motifs of the form G_3+_L_8–12_ (red sector), i.e. three-tetrads structures with less predicted stability compared to the previous category, follow a similar pattern. Conversely, the two-tetrad motif (green sector) show an opposite trend, occupying a larger fraction of OQs in those species with less canonical PQS motifs.

**Table 2.** OQs structural categories under K^+^ condition. Counts for the same motifs shown in Figure 3A and fold enrichments for all species, calculated as explained in Figure 3E (see Materials and Methods).

Genome	# all OQs	# G_3+_L_1–7_	# G_3+_L_8–12_	# G_2_L_1–12_	# Other	Fold enrich. G_3+_L_1–7_	Fold enrich. G_3+_L_8–12_	Fold enrich. G_2_L_1–12_	Fold enrich. Other
*human*	434 272	180 467	65 400	153 564	34 841	47.3	15.4	1.2	0.1
*mouse*	797 789	327 452	95 906	285 543	88 888	41.2	14.8	1.4	0.2
*zebrafish*	141 637	25 677	11 291	45 913	58 756	90.1	37.8	2.7	0.5
*Drosophila*	19 399	5262	3296	7966	2875	55.8	26.0	1.7	0.2
*C. elegans*	4144	1561	319	1911	353	173.4	56.3	3.4	0.1
*Saccharomyces*	103	7	3	59	34	21.0	9.1	4.5	0.4
*Leishmania*	17 343	7913	1802	6816	812	17.8	7.8	0.8	0.1
*Trypanosoma*	3236	635	288	1788	525	47.6	19.2	1.9	0.2
*Plasmodium*	173	51	4	50	68	154.6	12.1	18.8	0.4
*Arabidopsis*	2407	338	159	1241	669	338.0	79.5	3.9	0.3
*E. coli*	47	5	4	32	6	15.2	12.1	1.8	0.2
*Rhodobacter*	291	109	56	120	6	32.7	14.0	0.5	0.1

In fact, the proportion of observed two-tetrad G4s in particular increases upon PDS stabilization (green sectors in Figure 3A and B) for all genomes. Also, the total numbers of PQS motifs identified as OQs and the identified percentage of PQS out of the total genomic motifs increases in all species when comparing the K^+^ to PDS conditions (Figure 3B, C and Supplementary Figure S4), consistent with the small-molecule enabling more sensitive G4 detection. Overall, PDS treatment greatly increases the average assay sensitivity from 31% to 66%, since the small-molecule stabilization allows more PQS to be identified, and also to a lesser extent increases the average specificity from 81% to 85%, since OQs are scored using an increased threshold, which suppresses false positives.

In *mouse*, for instance, there is a general increase in all G4 categories (Figure 3D), but only the two-tetrad category shows a significant increase in the fold enrichment over random (from 1.4 to 1.8; *t*-test *P*-value = 10^–6^; Figure 3E).

Poly-G stretches, i.e. sequences consisting of 12 or more consecutive Gs, also appear to be prone to stably form G4s, as 83% of the ∼40 000 poly-G stretches combined across all species is identified as OQs in K^+^ conditions, with the percentage further increasing to 92% in PDS stabilizing conditions. The long stretch (∼427 nucleotides) of Gs present in the human genome in chromosome 2 (chr2:33141266–33141693) previously reported in Huppert (34) also displays OQs formation in PDS across the entire region, while in K^+^ OQs are detected just below threshold (% mismatches of 23).

We also observed an increased enrichment for the two-tetrad category in PDS versus in K^+^ of each respective species (see Table 2 for K^+^ and Supplementary Table S3 for PDS). Notably, the enrichment for the three-tetrad motifs (both G_3+_L_1–7_ and G_3+_L_8–12_) under K^+^ condition was very high for *Arabidopsis, C. elegans, Plasmodium* and *zebrafish* and low for *Saccharomyces, E. coli* and *Leishmania*, suggesting that PQS motifs can score differently across species (Table 2). The additional stabilisation induced by PDS, caused a higher enrichment for the three-tetrad G4 motif in *Saccharomyces* and *E. coli* (Supplementary Table S3). Given G4-seq is carried out on isolated single-stranded DNA, we reasoned that the observed differences must be due to species-dependent sequence effects within and around the G4 motif, which we discuss in the next section.

### Features of stable G4s identified

**Figure 4.**
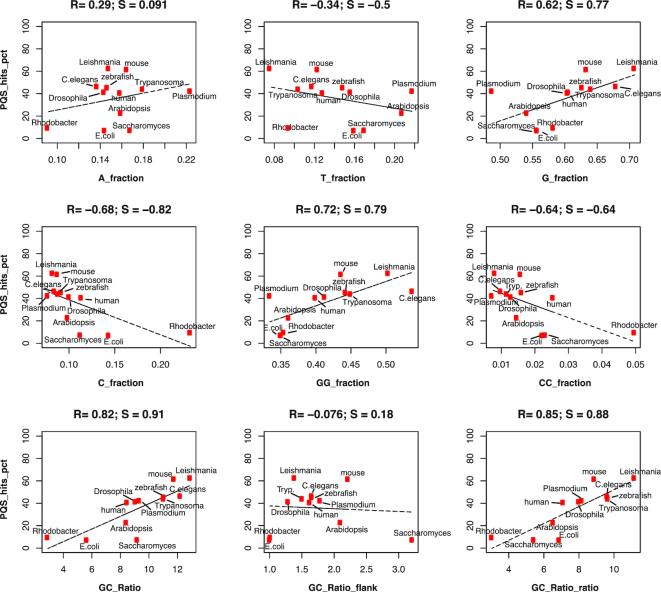
Dependency of overall PQS scoring from sequence features. Scatter plots highlighting the dependency of a sequence feature within the PQS motif (G_3+_L_1–12_), such as percentage of Gs (G_fraction) or the G/C ratio (GC_Ratio) on the x-axis and the genome-wide proportion of the same PQS motif identified as scoring OQs in K^+^ (PQS_hits_pct) on the y-axis. The linear regression for each pair is shown as a dashed line. The numbers at the top of each plot indicate respectively the Pearson correlation coefficient (*R*) and the Spearman correlation coefficient (*S*). Positive values indicate that the sequence feature has a positive association with PQS scoring proportion, i.e. that having higher values of this feature also determine higher likelihood of PQS to be identified as OQs. Abbreviation: Tryp. = *Trypanosoma*.

We next evaluated how particular sequence-related features, such as G4 loop sequence and G4 flank sequences, which influence G4 formation and stability, might explain the observed inter-species differences in G4 stability (Figure 2C). We considered the average measurement of a certain feature (e.g. G-richness) in all PQS across the 12 species and compared it to the percentage of all PQS scoring in each respective species (Methods). The PQS scoring proportion did not depend on overall GC content (R = -0.2) and was only marginally affected by PQS density (*R* = 0.35) (Supplementary Figure S5). Rather, the PQS scoring proportion showed, a strong dependency on G- and GG-richness (*R* = 0.62 and 0.72) and a negative correlation with C- and CC-richness (*R* = –0.68 and –0.64) (Figure 4), while T- and A-richness had a smaller effect (*R* = 0.29 and –0.34, respectively). G/C ratio, defined as G fraction divided by C fraction within the PQS motif, was an even stronger determinant of PQS scoring proportion (*R* = 0.82), with the degree of GC-richness in the flanking regions having no effect (*R* = –0.08) (Figure 4). Interestingly, these observations are in agreement with a recent multi-organism computational study reported by Burrows and co-workers (35). The C-, G-, T-, A-richness and G/C ratio were calculated within the PQS motifs, and the average PQS values for those are reported in Supplementary Figure S6. Notably, bacterial genomes, which showed a general absence of OQs in K^+^, are characterized by low G/C ratio within PQS motifs.

Sequence features, either individually or in combination with each other, can be used to predict the PQS scoring proportion by performing a linear model fitting. We also considered as additional predictive feature the G4Hunter score, which considers G- and C-richness and G/C asymmetry (18). Among all the features tested, G/C ratio, the G4Hunter score and the linear combination of G and C (or GG and CC) produced the best fitting (all R > 0.8; Supplementary Figure S7), confirming that G and C richness are the strongest determinant of G4 formation and stability. The negative effect of cytosines on G4 stability, assessed either as C-richness alone or in relation to G richness (G/C ratio and G4Hunter score), has been suggested for RNA (36–38) and for DNA (18,20). We have now observed and quantified this genome-wide across species, which explains the majority of PQS not scoring as stable G4s in bacteria genomes.

### Genomic location of OQs in different species

**Figure 5.**
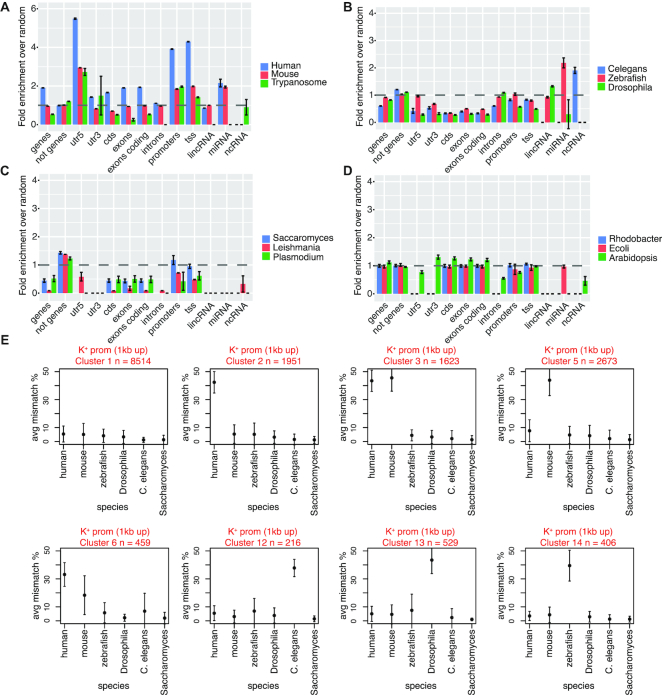
Enrichment of OQs in genomic regions and promoter co-occurrence cluster analysis. (**A**) Bar plot showing fold enrichment of OQs regions in different genomic regions, such as 5′UTRs, promoters, exons, non-coding RNAs, for human, mouse and Trypanosoma species. The fold enrichment is calculated, for each genomic feature independently, as the intersection of OQs with the feature, divided by the random overlap with the same feature, assessed after random permutation of OQs within the whole genome (Materials and Methods). (**B**) Same as A), but for *C. elegans*, zebrafish and Drosophila. (**C**) Same as A), but for Saccharomyces, Leishmania and Plasmodium. (**D**) Same as A), but for Arabidopsis, Rhodobacter and *E. coli*, Species have been grouped according to their fold enrichment profile, to have similar enrichments in the same graph. Bars represent mean fold enrichment (*k* = 3 independent randomization) with SEM (Supplementary File S1). (**E**) Average mismatch percentage values (y-axis) calculated in promoter regions of different clusters identified by the promoter cross species OQs conservation analysis in K^+^ (Materials and Methods). The six most related eukaryotic species (x-axis) are considered in this analysis. Titles above each plot indicate cluster name and number of promoters in each cluster.

To understand how OQs distribute with respect to key genomic structural elements, we downloaded gene annotations for all species and counted the OQs (considering all three categories) occurring in each region (Supplementary Table S4, Materials and Methods). The distribution of G4s showed considerable variation across species (Supplementary Figure S8). Enrichment or depletion of OQs in different regions was determined by comparison to random occurrence (Materials and Methods) within the same species (Figure 5A–D and Supplementary File S1). The most striking observation was for *human, mouse* and *Trypanosoma*, where we observed a strong enrichment of OQs at gene promoters (1 kb upstream of TSS) and in 5′UTR regions, with *human* having the strongest enrichment (Figure 5A). In contrast, other eukaryotic species (*C. elegans, zebrafish* and *Drosophila*) showed depletion at these and other (e.g. exons, 3′UTR) intragenic regions (Figure 5B). *Saccharomyces, Leishmania* and *Plasmodium* genomes similarly showed depletion at intragenic regions (Figure 5C), but differently from the previous group (*C. elegans, zebrafish* and *Drosophila*) did not exhibit enrichment at non-coding RNAs. The last group, *Rhodobacter, E. coli* and *Arabidopsis*, did not show enrichment or depletion of OQs at any genomic regions.

### Cross species analysis of OQs occurrence at promoter regions

The incidence of G4s at gene promoter regions is relevant for hypotheses linking G4 formation to gene transcriptional activity (21,39–42). We considered OQs at promoters (1 kb upstream of TSS) in the 6 most closely related eukaryotic species (*human, mouse, Drosophila, zebrafish, C. elegans, Saccharomyces*), of those we studied, to evaluate any cross-species co-occurrence patterns (Methods, Supplementary File S2). A proper analysis of G-quadruplex evolutionary conservation was not the goal of this study and would require a different choice of organisms. However, inspecting multiple genomic maps can still provide insights into G4 promoter occurrence, and help generating hypotheses about similarities and differences of the G4-mediated transcriptional control in different species. Therefore, as part of this analysis we did not consider exact sequence conservation, as the genomes of these species are not closely conserved.

Hierarchical clustering analysis, where we analysed the signal at promoter of orthologues (Materials and Methods), showed 8 interesting co-occurrence patterns present in at least 200 promoters (Figure 5E). More clusters could be observed (Supplementary Figure S9) but were relatively low in abundance (less than 200 promoters per group), therefore we restricted the analysis to highly abundant patterns.

Many gene promoters did not have OQs in the promoters of any species (*n* = 8514, cluster 1; Figure 5E and Supplementary Figure S10), but interestingly a consistent number had OQs only in *human* and *mouse*, either specifically in one species (*n* = 1951 and *n* = 2673 for *human*- and *mouse*-specific, respectively; clusters 2 and 5) or in both species (*n* = 1623, cluster 3). On the other hand, some promoters exhibited OQs with higher mismatch values, hence higher predicted stability, in *human* compared to *mouse* (*n* = 459, cluster 6) (Figure 5E). Interestingly, a direct comparison of the mismatch values in *human* and *mouse* promoters highlights some similarities in OQs formation at both promoters (over 2000 regions) but also substantial differences, with over 5300 related promoters exhibiting OQs (i.e. mismatches ≥ 25%) in only one species (Supplementary Figure S11). A lower but substantial number of promoters had OQs only in one species, either *C. elegans* (*n* = 216, cluster 12), *Drosophila* (*n* = 529, cluster 13) or *zebrafish* (*n* = 406, cluster 14) (Figure 5E). Detailed heat-maps of the 8 major promoter OQs co-occurrence patterns can be inspected at Supplementary Figure S10 and Supplementary File S2. Other combinations, such as OQs at conserved promoters in multiple species (e.g. *human, mouse* and *Drosophila* or *human, mouse* and zebrafish) also existed (Supplementary Figure S9, clusters 9 and 17), but in lower number.

We performed gene ontology and KEGG pathways enrichment analysis to infer if any particular functional category was enriched in the 8 major cluster groups (Methods, Supplementary File S3). Consistent with previous reports (20,21,43), we observed G4s in *human* to be strongly associated with regulatory regions of cancer-related genes and somatic copy number variations. In particular, promoters having OQs in both *human* and *mouse*, but not other species, displayed enrichment in cancer pathways as well as in genes from the cancer gene catalogue COSMIC (83 genes from the cluster 3, hypergeometric test *P*-value <0.01 for the cosmic gene enrichment within the cluster) (Supplementary File S3, *all_clusters_KEGG* tab). Regarding the *human/mouse* specific OQs promoter (cluster 3), we also noted that pathways involved in development, neurological activity and cardiac function were enriched. These genes were also strongly enriched for transcriptional regulation and developmental processes (Supplementary File S3, *all_clusters_BP* tab), whereas *human* only OQs-containing promoters (cluster 2) were enriched specifically in amino acid transport pathways, protein sumoylation and protein folding, to name a few.

## DISCUSSION

The G4-seq approach for sequencing G-quadruplexes exploits specific properties of G4 folding by comparing sequencing outputs in conditions that stabilise folded G4s with sequencing under conditions that do not stabilise G4s (20). Our second-generation approach employed here applies the same general principles but provides improved coverage at GC-rich and G4 regions. This improvement was particularly advantageous for establishing G4 maps in challenging, GC-rich genomes such as *Leishmania* and *Rhodobacter*, and for obtaining accurate information at GC-rich regions in the *human* and *mouse* genomes that, otherwise, would lack sufficient coverage. In our original G4-seq based *human* genome map, 20% of the identified OQs could not be ascribed to a defined G4 motif, which represents the false positive rate of the method. Our improved method uses Li^+^ instead of Na^+^ in the reference sequencing run (*Read-1*), leading to a lower basal level of G4 stabilisation. This change reduced the apparent false positive rate to just 8%. Another improvement was the ability to separate proximal G4 peaks by adopting a smaller window in the scoring pipeline to increase spatial resolution, which increased G4 peak resolution.

A striking observation based on the multi-species OQ maps is the strong depletion of G4s observed in bacterial genomes and in yeast (Figure 2C). Previous computational studies have made predictions about G4 formation leading to suggestions about potential regulatory roles on transcription in bacteria (44,45) and highlighted the effects of G4s in causing genetic instability in yeast (46). At first glace, our data actually aligns with a recent study (47) that experimentally investigated RNA G-quadruplex formation in bacteria and suggested that G4s may have been deselected through evolution. However, a higher proportion of predicted G4 motifs were detected as OQs in bacteria and yeast upon inclusion of the small molecule PDS (Figure 2C), suggesting some potential to form G4s. Further stabilization of the G4 during specific cellular processes, e.g. by protein interaction or in specific genetic backgrounds, could enable G4 formation and induce associated cellular effects. For example, functional effects of G4s have been specifically observed in *FANCJ* mutants both in *C. elegans* (48) and *human* cells (49), and after G4 stabilisation by small molecules or genetic *PIF1* deletion in yeast (50).

We found that key sequence features, such as G and C richness, and the G to C ratio within the PQS motifs, can explain the global depletion or abundance of G4s observed in different species in K^+^ (Figure 4). As this is a global correlation analysis, predictions of individual G4s would need more sophisticated machine-learning approaches, as recently exemplified for the *human* genome (17). Another striking outcome of our study is the difference between species with regard to where in the genome G4s are positionally enriched. We observed strong G4 enrichment at promoter and TSS regions specifically in higher species such as *human* and *mouse*. Interestingly, the *Trypanosoma* genome also showed a similar enrichment pattern, despite being evolutionarily distant from *mouse* and *human*, which may reflect similarities in the G4 biology. Thus, the link between G4s and transcriptional control is worthy of further exploration in the future. Other vertebrates show, instead, a mild depletion at promoter regions, or even a strong reduction at any intragenic features, such as exons and UTRs (Figure 5A–D). In Ding *et al.* (35), where they studied G-quadruplex formation for hundreds of microorganisms with a focus on the bacterial orders Deinococcales and Thermales, Thermales does not show G4 density near the TSS while Deinococcales does. In our data, on the other hand, Trypanosoma shows a genomic pattern of G-quadruplex occurrence similar to human and mouse. These observations, take together, suggest that species close in evolution can display fundamental differences in G4 localization and function, and vice versa distant species can present intriguing similarities. This interesting aspect will benefit from future evolutionary studies.

Clustering analysis of promoter co-occurrence for the six higher eukaryotic species revealed patterns of species-specific occurrence, such as the OQs found exclusively in *mouse* and *human*, or OQs unique to a single species. Preliminary enrichment analysis revealed processes or pathways associated with particular sub-classes of promoters, such as cancer genes for the promoter OQs specific to both *mouse* and *human*. A related observation was previously reported for *human* by computational analysis (43), *in vitro* (20) and in cells (21). This association suggests a specific role for G4 at promoters of oncogenes in mammals.

Evaluating where G4s form within the genome provides insights into how they may be exploited functionally, for example during development (43,51), and how they may be targeted for the treatment of conditions such as cancers (52,53). Our study has identified over 3.5 million G4s across 12 species, which constitutes the largest experimental G4 dataset to-date. This large dataset may enable computational and machine-learning approaches to better elucidate sequence and structural features for G4 formation, leading to improved predictors. We anticipate the G4 maps will be a valuable resource for the scientific community to probe and understand biology that might involve G4s.

## DATA AVAILABILITY

Raw and processed data for all 12 species from this study have been submitted to the NCBI Gene Expression Omnibus (GEO; http://www.ncbi.nlm.nih.gov/geo/) under accession number GSE110582. A detailed explanation of all the processed files is provided in Supplementary File S4.

## Supplementary Material

Supplementary Data
