# Supplementary material for: Whole genome experimental maps of DNA G-quadruplexes in multiple species
Source: Nucleic Acids Res. 2019 Mar 20;47(8):3862–74. doi: 10.1093/nar/gkz179 (PMC6486626; doi:10.1093/nar/gkz179)
Supplement: Supplementary Data [file gkz179_supplemental_files.zip › Supplemental_Material_NAR_revised_20190304.pdf]

SUPPLEMENTARY DATA

Whole genome experimental maps of DNA G-quadruplexes in multiple species.

Marsico et al.

Contents:

- **The improved G4-seq method.**
- **Supplementary Figures S1 through S16.**
- **Supplementary Tables S1 through S4.**
- **Supplementary References.**

The improved G4-seq method

Limitations of the previous G4-seq method

G4-seq relied on two sequencing runs of PCR-amplified genomic DNA: the first (*Read-1*) performed in Na^+-rich buffer conditions, where G4s are not greatly stabilized, and a second re-sequencing run (*Read-2*) performed in K^+ or after addition of the potent G4-stabilising ligand pyridostatin (51) (Na^++PDS conditions), where G4s are selectively stabilized. When no G4 is formed, sequencing reads under G4-stabilising conditions displayed no or very low mismatches when compared to the reference run in *Read-1*. Conversely, a high number of mismatches between the two runs were identified when a G4 structure is formed and stabilised by either K^+ or PDS, with G4s of higher predicted stability usually displaying higher number of mismatches (20). To perform genomic assessment, an averaging window of 150 nucleotides (nt) was considered in the analysis when measuring mismatch level between *Read-1* and *Read-2* at regions where *Read-1* reads were used to align to the reference genome. Mismatch percentage levels were finally used as a proxy for G4 forming potential, and putative stability, in genomic context (17,20).

The most important limitation of the original G4-seq approach was lack of sequencing coverage in certain GC-rich regions of the genome, which prevented analysis of potential G4 containing regions. To quantify this effect, we analysed in-depth chromosome 1 (chr1) from G4-seq and divided it into over 9 million overlapping windows of 50 nt: 3.6% of those did not have coverage. We then focused the analysis on only those windows having GC content over 70% (*n = 105,000*): a greater fraction corresponding to 11.3% (~12 k windows) did not have coverage. The percentage of windows with no coverage further increased to 15.3% (5,878 windows) when considering the subset of GC-rich windows containing the putative G4 forming motif $G_{3+}L_{1-12}$ (total of *38,329*). Additionally, a considerable number (over *26,000*, 7.4% of total) of putative G4 structures (PQS) of the form $G_{3+}L_{1-7}$ did not have coverage on a genome-wide scale. In light of this, we reasoned that the coverage problems are mostly due to: 1) high GC richness that hampers efficient PCR amplification; 2) presence of G4s that interferes either

with PCR amplification prior to sequencing or with sequencing in Na^+, where the G4 itself could be intrinsically stable.

This lack of coverage makes it impossible to estimate the mismatch level, which is the indicator of the G4 forming potential (17,20), in potentially interesting genomic regions (see examples in Supplementary Figure S12). This would pose significant limitations for the study of genomes that are either GC- or G4-rich, such as Leishmania and Rhodobacter (Table 1). Problematic PCR amplification during library preparation at GC-rich regions (55) and the high stability of certain G4s even in Na^+-rich sequencing conditions have been previously observed (7,56,57).

Another limitation of G4-seq was the broadness of the identified OQs motifs: the reported median size was 255 nt (Supplementary Figure S13A), significantly larger than a typical, single G4 motif (20-50 nt). Low resolution does not imply low accuracy: when considering the summit (i.e., the region with highest value within a peak) of the mismatch percentage track, the PQS motif relative to the G4 identified was in a proximity of 15-30 nt from the summit itself, as exemplified in Supplementary Figure S13B. However, low resolution implies that two G4 motifs in close proximity will not be resolved, hence generating a broader peak (Supplementary Figure S13C).

Lastly, the PDS stabilising conditions performed in the presence of Na^+ instead of K^+, which was due to experimental constraints in the sequencing protocol, could potentially not display the full range of G4 motifs that can be formed/stabilized by the addition of the ligand, as K^+ is the preferred cation for G4 stability (Davis et al. 2004). In fact, only 63% of the canonical PQS motif $G_{3+}L_{1-12}$ was identified as OQs under the PDS condition in G4-seq.

Characterization of the improved sequencing methodology

Key limitations of G4-seq included lack of sequencing coverage in certain GC-rich regions of the genome (Supplementary Figure S12) and limited resolution for the identified OQs motifs (Supplementary Figure S13), which have been extensively described previously (17,20) and are further detailed in this Supplementary Data.

Instead of Na^++PDS conditions; mismatch analysis performed in smaller averaging windows of 50 instead of 150 nucleotides, improving the resolution of OQs identification; not shown in the diagram, PCR-free library preparation strategy (details in the main text).

To improve these issues, we adapted G4-seq in the following ways: 1) carry out the first sequencing run (*Read-1*) in Li^+ instead of Na^+, to ensure even less G4 stabilisation in the control run; 2) PCR-free library preparation method, to avoid PCR-induced coverage bias; 3) K^++PDS (57) as strong stabilizing conditions (*Read-2*), to increase the sensitivity of G4 detection; 4) new data analysis pipeline to improve scoring/localization of OQs (Figure 1B). To assess in depth all the introduced methodological changes, we sequenced and analysed chromosome 1 (Chr 1) of the *human* genome, which contains greater than 60 k PQS of the form $G_{3+}L_{1\text{-}12}$ over 250 Mb of sequence (Methods). Li^+ is a weak stabiliser of G4s and therefore *Read-1* is now less likely to encounter stable G4s. Consistent with this, we noted that sequencing in Li^+ greatly improved the fraction of PQS sites with non-zero coverage, as compared to Na^+, especially for G4 motifs with loops shorter than 4 nucleotides, for both the PCR and PCR-free protocols (Supplementary Figure S14A). PCR generates coverage bias in whole genome sequencing (55,58), much of which can be removed using a PCR-free protocol. Indeed, PCR-free libraries substantially improved coverage in regions with GC-content over 70%, including those containing PQS motifs (Supplementary Figure S14B). This improvement is particularly important when analysing more GC-rich genomes. Under the K^++PDS G4 stabilizing condition, we identified a much greater number of OQs (hit scoring regions) that constituted 84% of the ~706 k PQS of the form $G_{3+}L_{1\text{-}12}$, as opposed to only 63% when using Na^++PDS. Thus, the higher stabilization offered by the K^+ cation improves the sensitivity of detecting expected G4s. Finally, the new scoring method uses windows of 50 nt, as opposed to 150 nt, when averaging the mismatch values (Methods). The total number of PQS ($G_{3+}L_{1\text{-}7}$) scoring as OQs is similar in both cases (Supplementary Figure S15A), but the resolution is much better, as indicated by the smaller OQs average size (Supplementary Figure S15B, D, E). Crucially, using the 50 nt window gives a lower number of OQs that comprise multiple PQS motifs compared to 150 nt (Supplementary Figure S15C), since G4 motifs in close proximity are better resolved than before (see example in Supplementary Figure S15F). Specifically, we estimated that 33% of

PQS motifs ($G_{3+}L_{1\text{-}7}$) show two distinct peaks in the mismatch profile, as exemplified in Supplementary Figure S16.

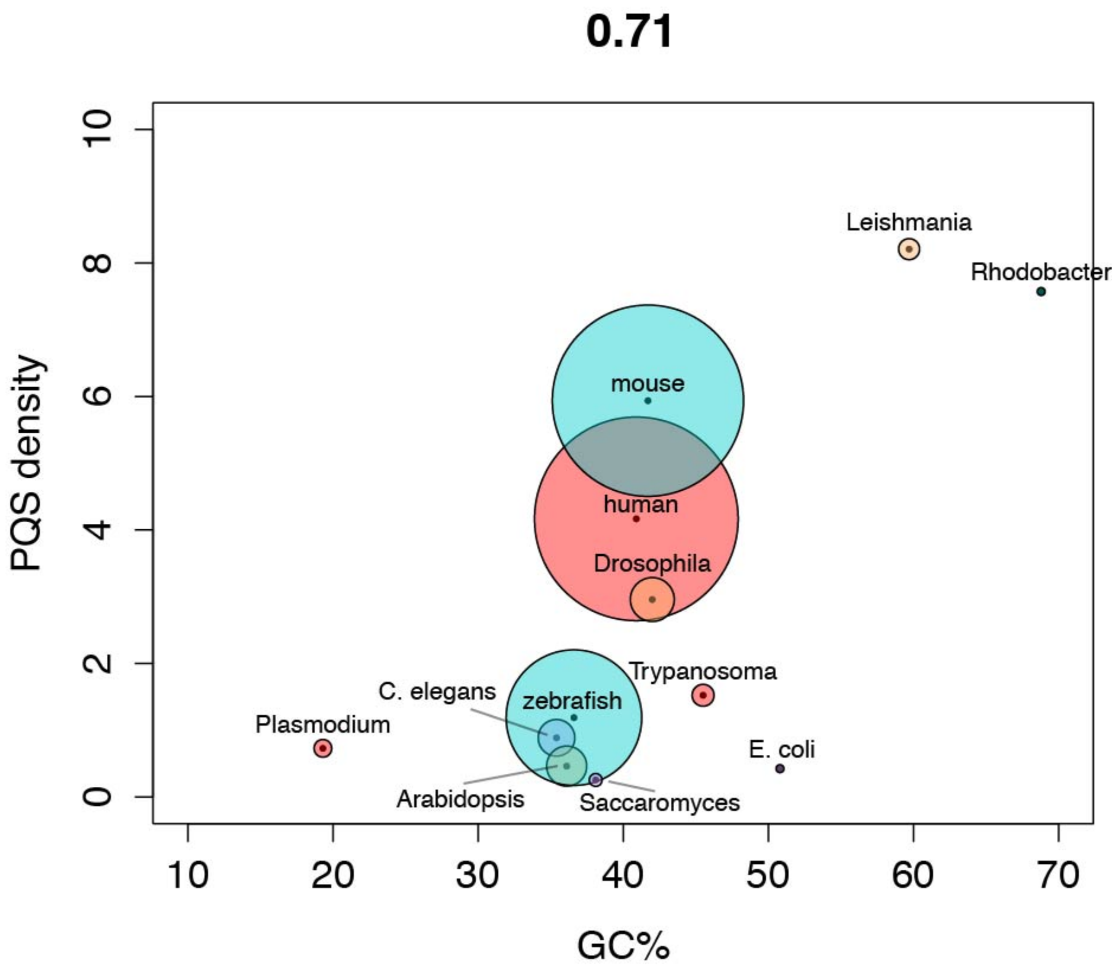

Supplementary Figure S1: Summary plot of GC richness and PQS density in all the 12 genomes. Circle size is proportional to the log of the genome size in bp. X-axis: overall genome GC content; y-axis: PQS density per kb, measured as the total size of $G_{3+}L_{1-12}$ PQS motifs per kb normalized by genome size (*1000 x PQS_total_size / (2 x genome_size)*).

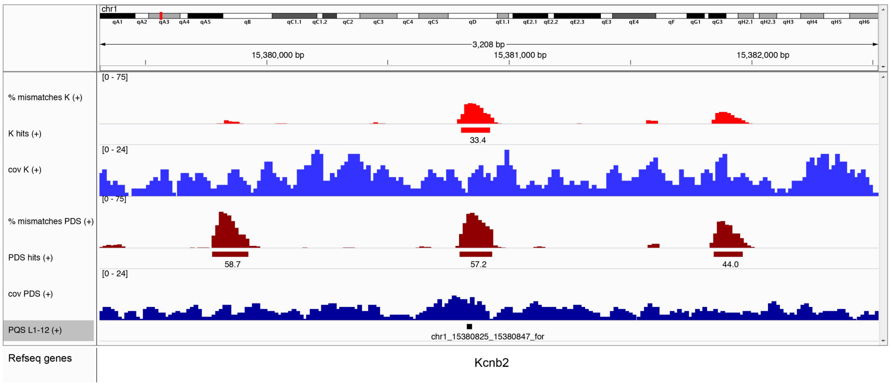

Supplementary Figure S2: IGV genome browser view of mismatch and coverage tracks and OQs intervals for K$^+$ and K$^+$+PDS conditions in mouse. Legend from top to bottom: Red peak track is the mismatch percentage in K$^+$ condition, forward strand. Red interval track is the OQs identified as hits in K$^+$, forward strand. Blue peak track: coverage in K$^+$, forward strand. Brown peak track is the mismatch percentage in K$^+$+PDS condition, forward strand. Brown interval track is the OQs identified as hits in K$^+$+PDS, forward strand. Blue peak track: coverage in K$^+$+PDS, forward strand. Blue interval track: PQS motif ($G_{3+}L_{1-12}$) on the forward strand. The mismatch percentage track in PDS (brown peaks) often shows higher mismatches than the one in K$^+$ (red peaks). All tracks are relative to mouse (*mm10*).

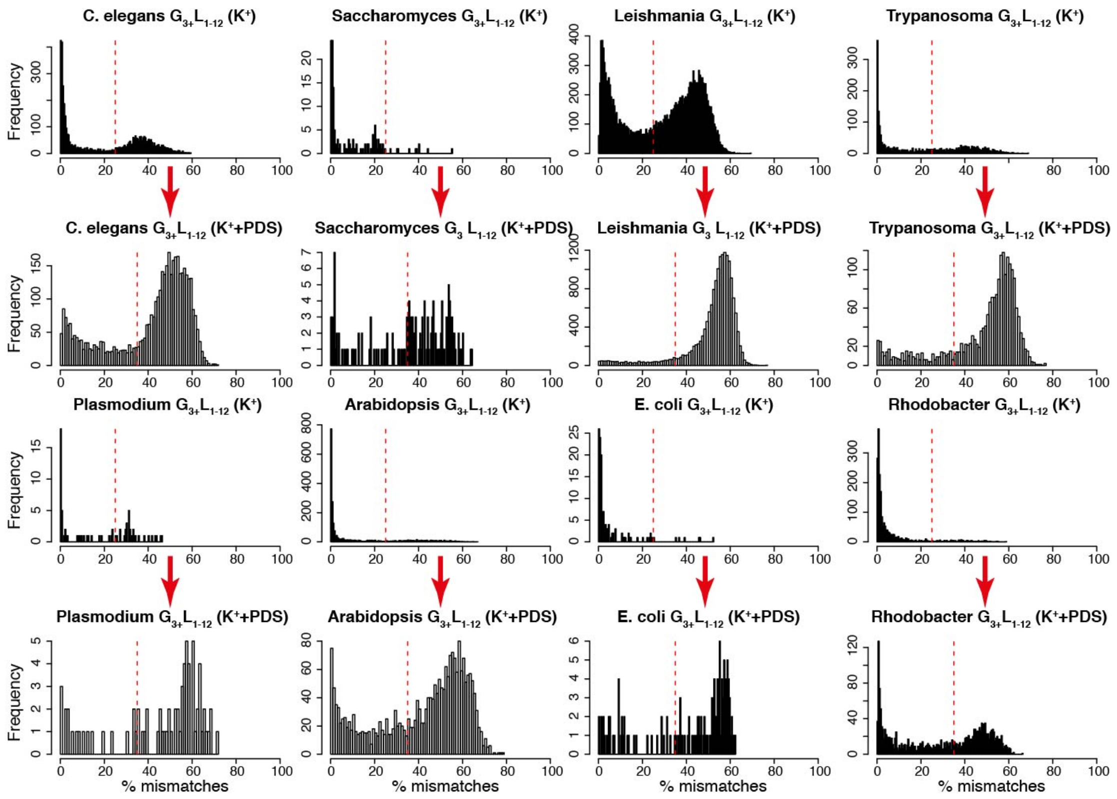

Supplementary Figure S3: Mismatch distribution for all PQS of the form $G_{3+}L_{1-12}$. Histograms showing the distribution of mismatch percentage levels for the $G_{3+}L_{1-12}$ PQS motifs; x-axes: mismatch percentage; y-axes counts. Top 2 rows: K^+ and PDS condition, respectively, for the indicated species labelled above each graph: C. elegans, Saccharomyces, Leishmania and Trypanosoma (left to right). Bottom 2 rows: K^+ and PDS condition, respectively, for the indicated species labelled above each graph: Plasmodium, Arabidopsis, E. coli and Rhodobacter. The right shift in PDS compared to K^+ towards higher mismatch percentage values is visible in each species.

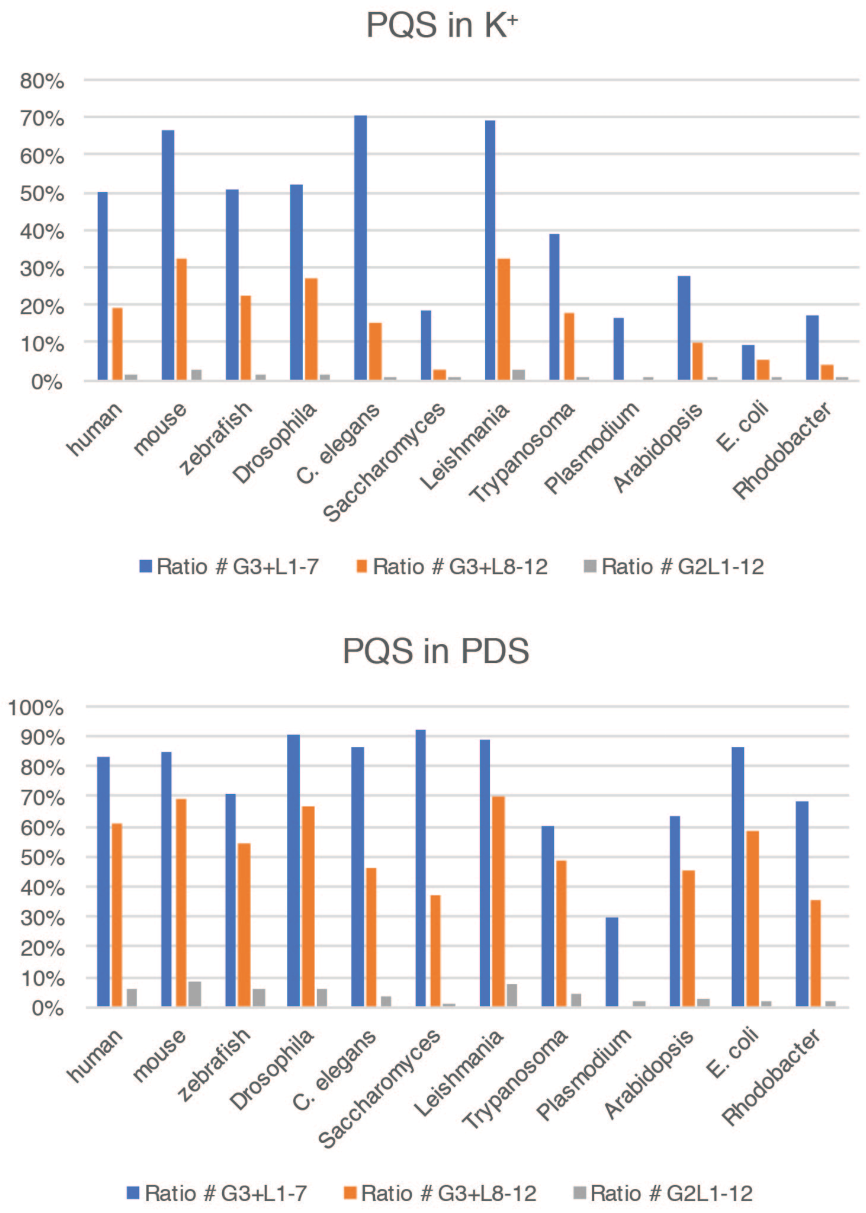

Supplementary Figure S4: Percentage of PQS motifs identified as OQS. Bar plots representing the percentage of PQS motifs from the different categories (indicated below in the plot legends) identified as OQs out of the total motifs present in different genomes (Supplementary Table S2). Top: K^+ condition; bottom: K^++PDS condition.

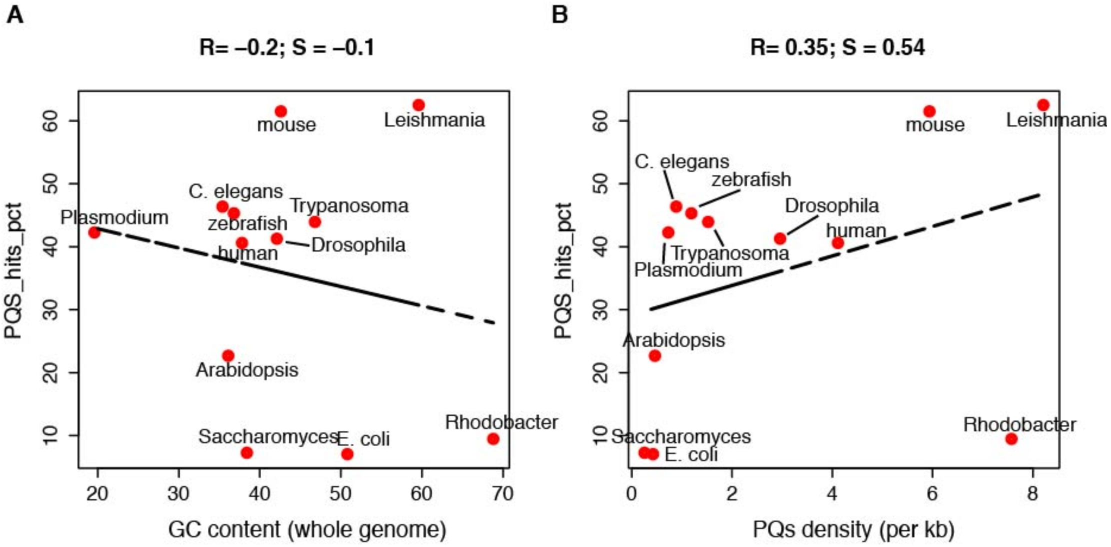

Supplementary Figure S5: Proportion of PQS scoring as a function of GC content and PQS density for the 12 genomes. A) Scatter plot indicating the dependency between overall GC content in the genome (x-axis) versus the percentage of PQS ($G_{3+}L_{1-12}$) identified as OQs (PQS_hits-pct, y-axis) for the 12 species, shown in red and labelled. The linear regression fit is shown as dashed line. **B)** Same as in A), but showing PQS density on the x-axis, calculated as the number of $G_{3+}L_{1-12}$ PQS motifs per kb. Titles above each scatter plot: R is the Pearson correlation coefficient; S is the Spearman correlation coefficient

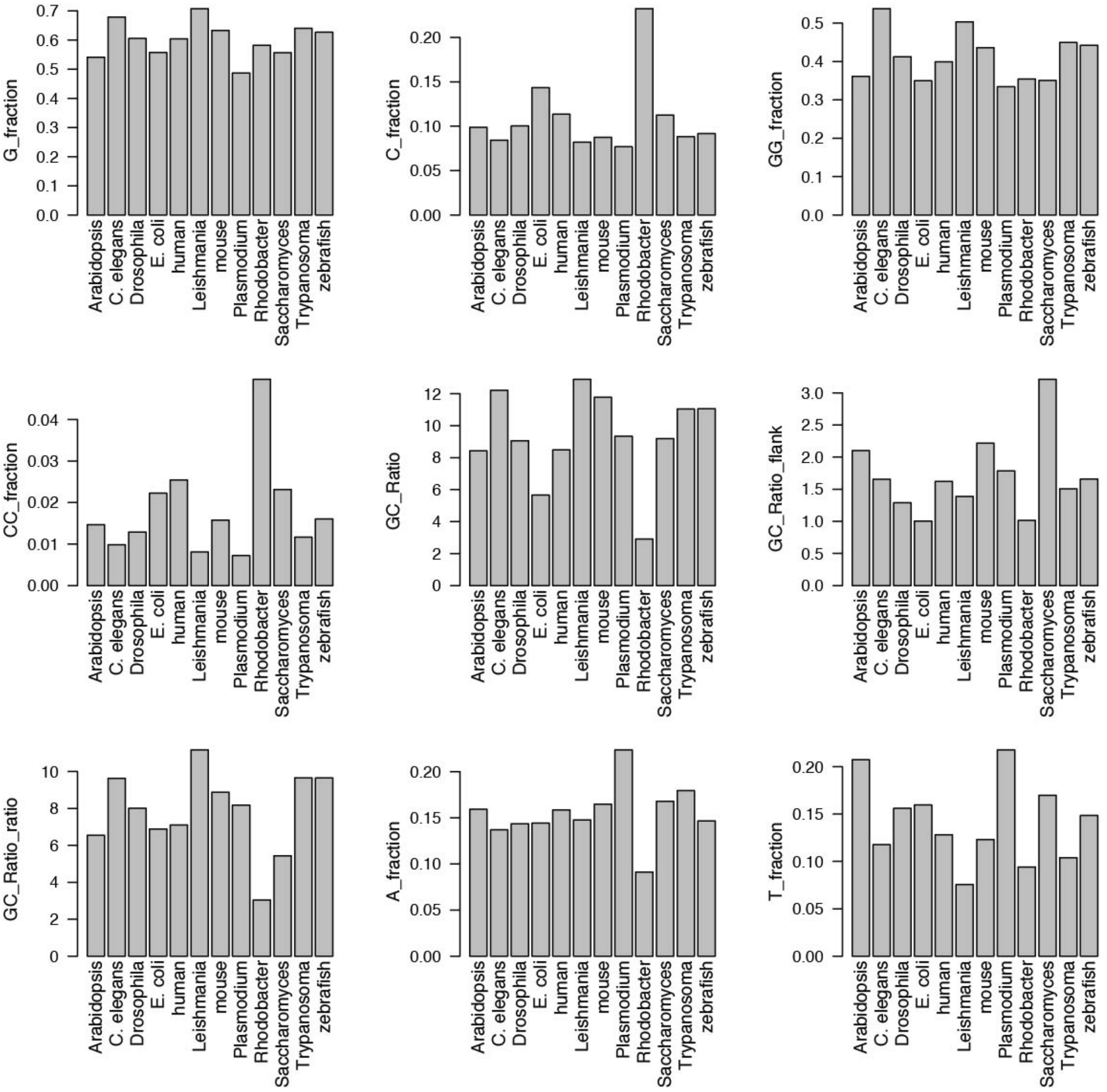

Supplementary Figure S6: Visualization as bar graph of the average PQS sequence features in each genome. Bar graphs showing the detailed values for each sequence features in all 12 species shown in Figure 4. X-axes: species names; y-axes: average value for the indicated sequence feature across all PQS motif ($G_{3+}L_{1-12}$).

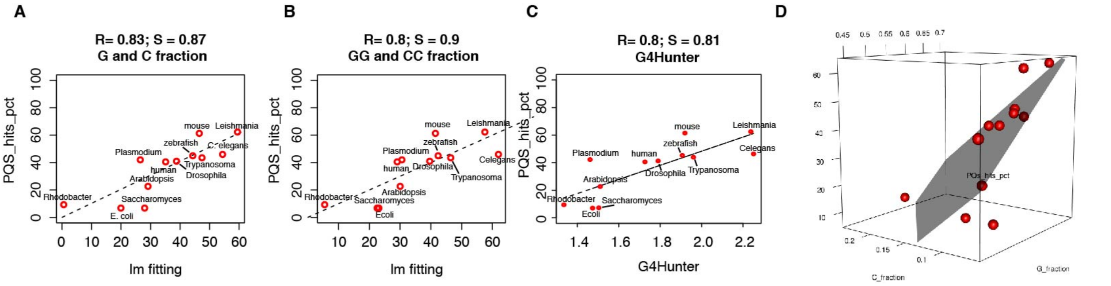

Supplementary Figure S7: Regression models to predict PQS scoring proportion. A) Fitting via linear model using G and C fraction as independent predicting variables, and the percentage of $G_{3+}L_{1-12}$ PQS identified as OQs as predicted variable (y-axis). Fitted model is shown as dashed line, and correlation between model fitting and observed value shown in plot title (R = Pearson correlation coefficient; S = Spearman correlation coefficient). **B)** As in A), using GG and CC fraction as predicting variables in the linear model fitting. **C)** as in A), using the G4Hunter scoring values as predicting variable. **D)** Representation through 3D plot of the linear regression model shown in A), with the fitted model shown as a plane: ax+by = 0, with x = C fraction and y = G fraction.

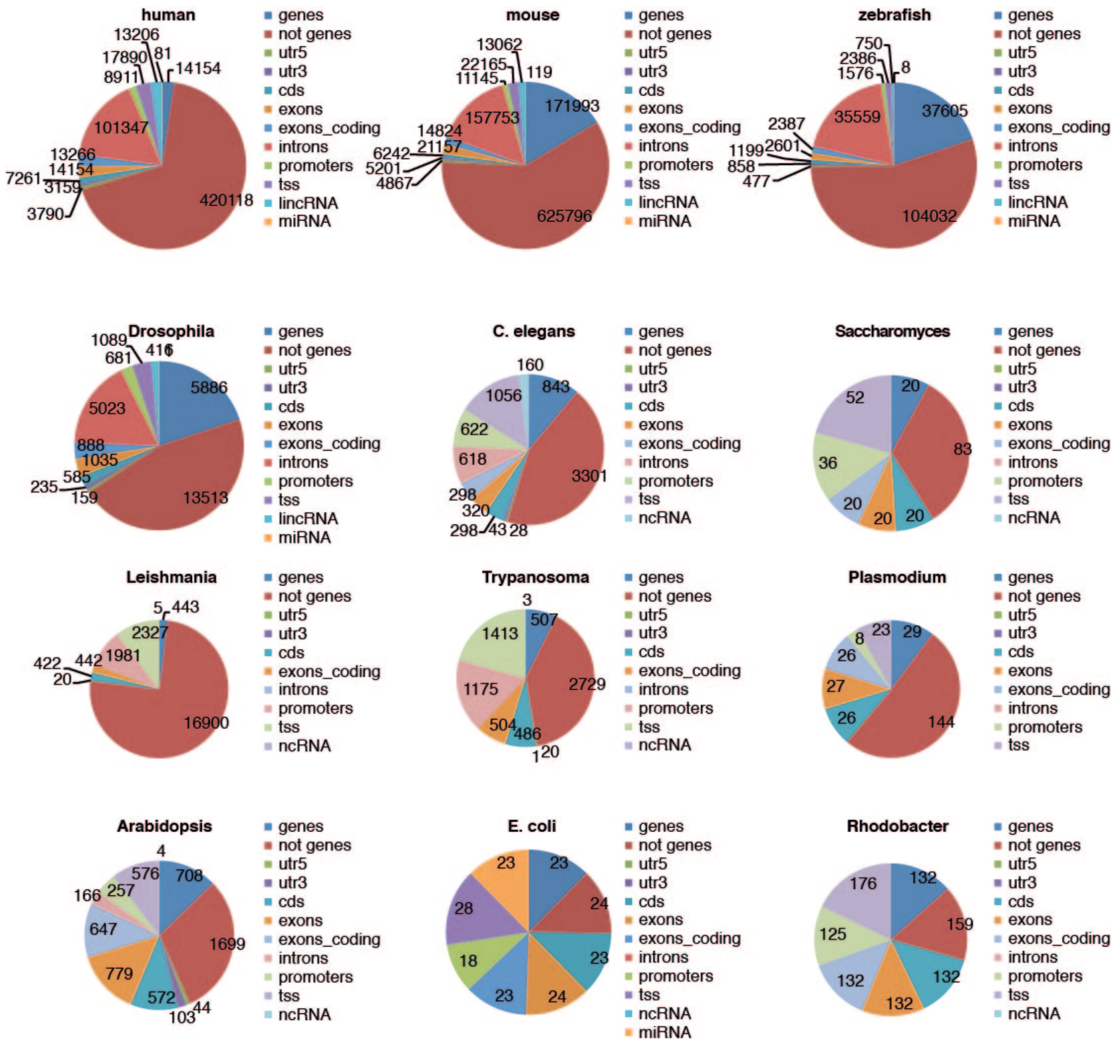

Supplementary Figure S8: Pie charts visualizing OQs distribution in different genomic regions from the respective annotation files in 12 species for the K+ condition. Counts for the number of OQs overlapping each genomic feature (e.g., genes, utr5, exons, etc; see legend next to pie charts) in the 12 different species, corresponding to the fold enrichment values shown in Figure 5A-D.

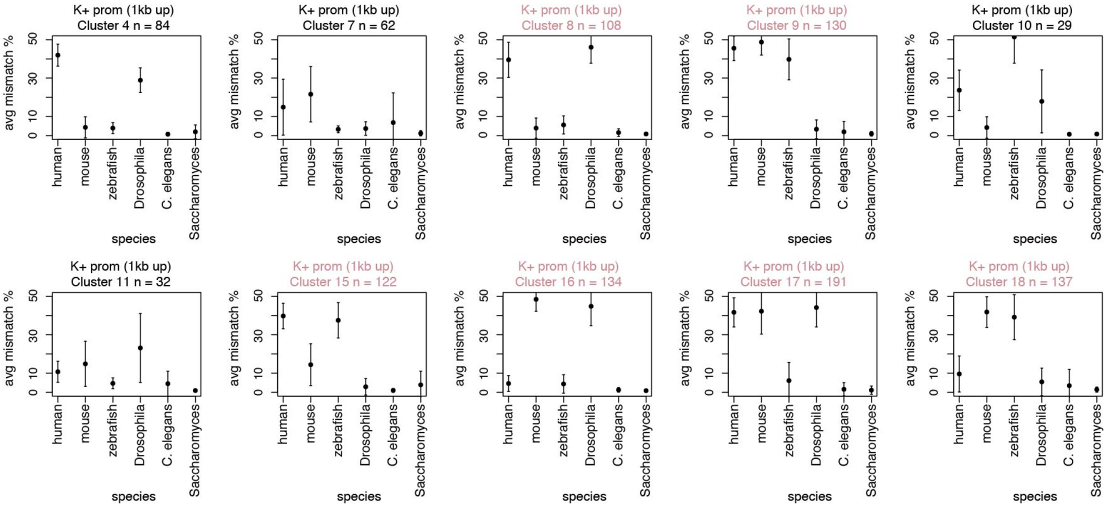

Supplementary Figure S9: Average mismatch levels for promoter classes with less than 200 items derived from clustering analysis of the promoter OQs co-occurrence. Average mismatch percentage values (y-axis) calculated in promoter regions of different clusters calculated in the promoter cross species OQs conservation analysis in K^+ (Methods). The 6 most related eukaryotic species (x-axis) are considered in this analysis. Titles above each plot indicate cluster name and number of promoters in each cluster.

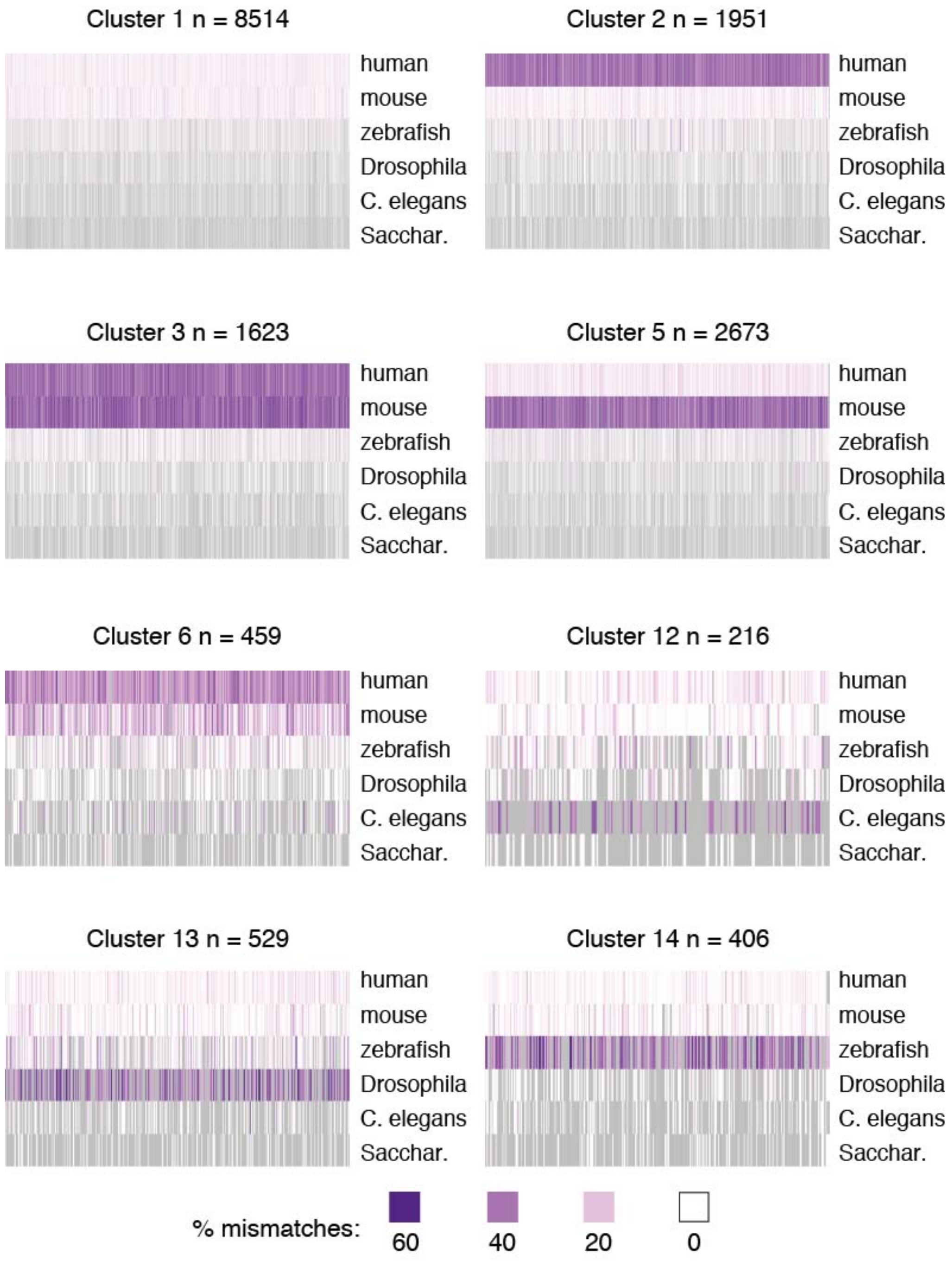

Supplementary Figure S10: Heat-maps for promoter classes with more than 200 items derived from clustering analysis of the promoter OQs co-occurrence. The average mismatch values for the same 8 most abundant clusters are shown in Figure 5E. Colour coding legend shown at the bottom, grey indicates missing values (i.e., orthologues of the human promoter not found in that species). Abbreviation: Sacchar. = Saccharomyces.

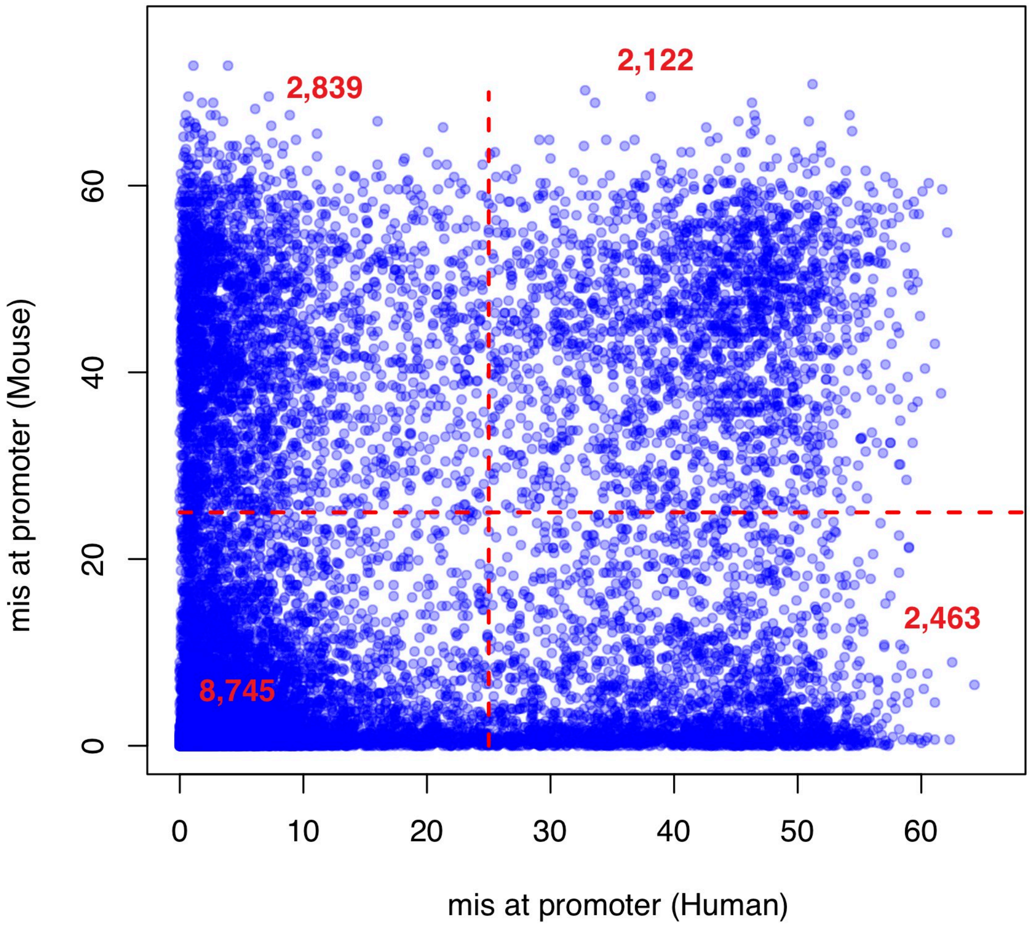

Supplementary Figure S11: Correlation between OQs at promoters of mouse and human. Scatter plot showing the correlation of mismatch values (mis, expressed in percentage) in human promoter (x-axis) and orthologue mouse promoter (y-axis). Total human promoters: 24,164; total human and mouse orthologue promoters: 16,169. Red text numbers indicate the number of genes in each respective quadrant separated by the dotted red lines. Red lines are drawn at the value of 25%, corresponding to the threshold used for OQs identification.

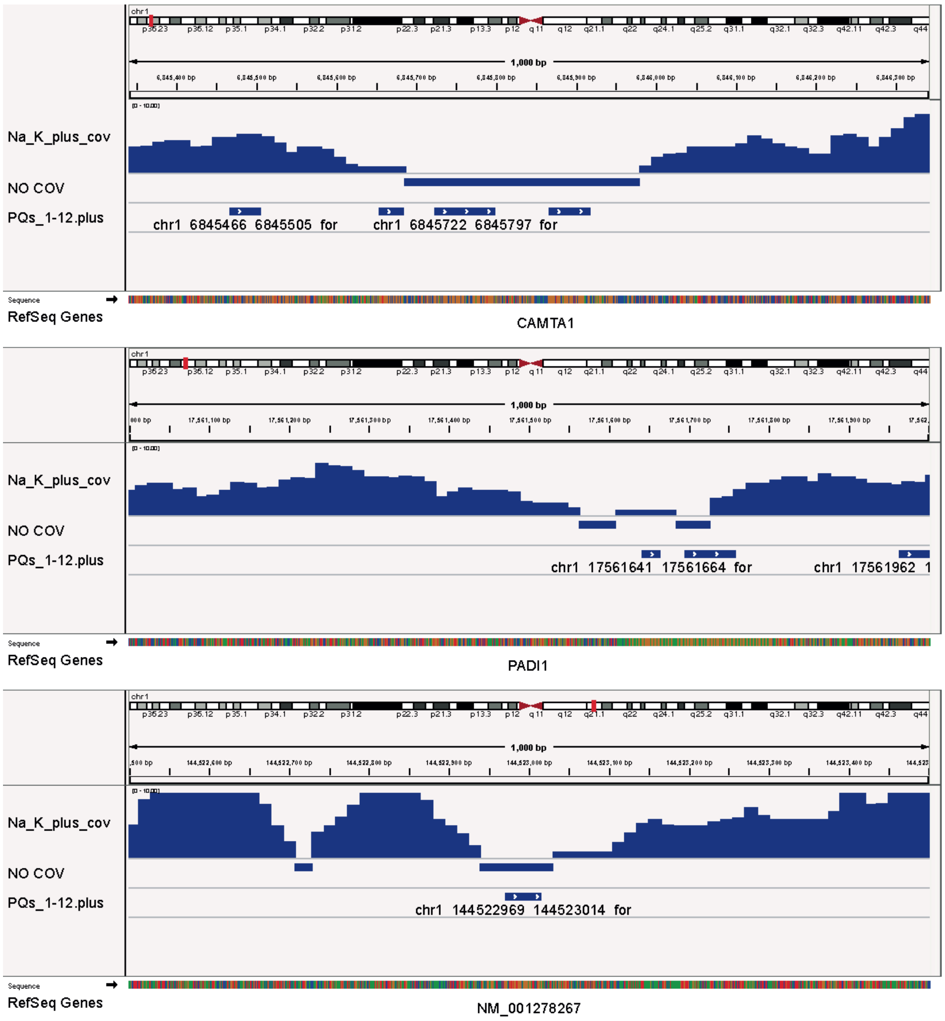

Supplementary Figure S12: IGV genome browser view of regions with no coverage in G4-seq. Blue tracks represent coverage tracks on the forward strand ("Na_K_plus_cov"); "NO COV" intervals represent regions with no coverage; "PQs_1-12_plus" intervals represent PQS of the form $G_{3+}L_{1-12}$.

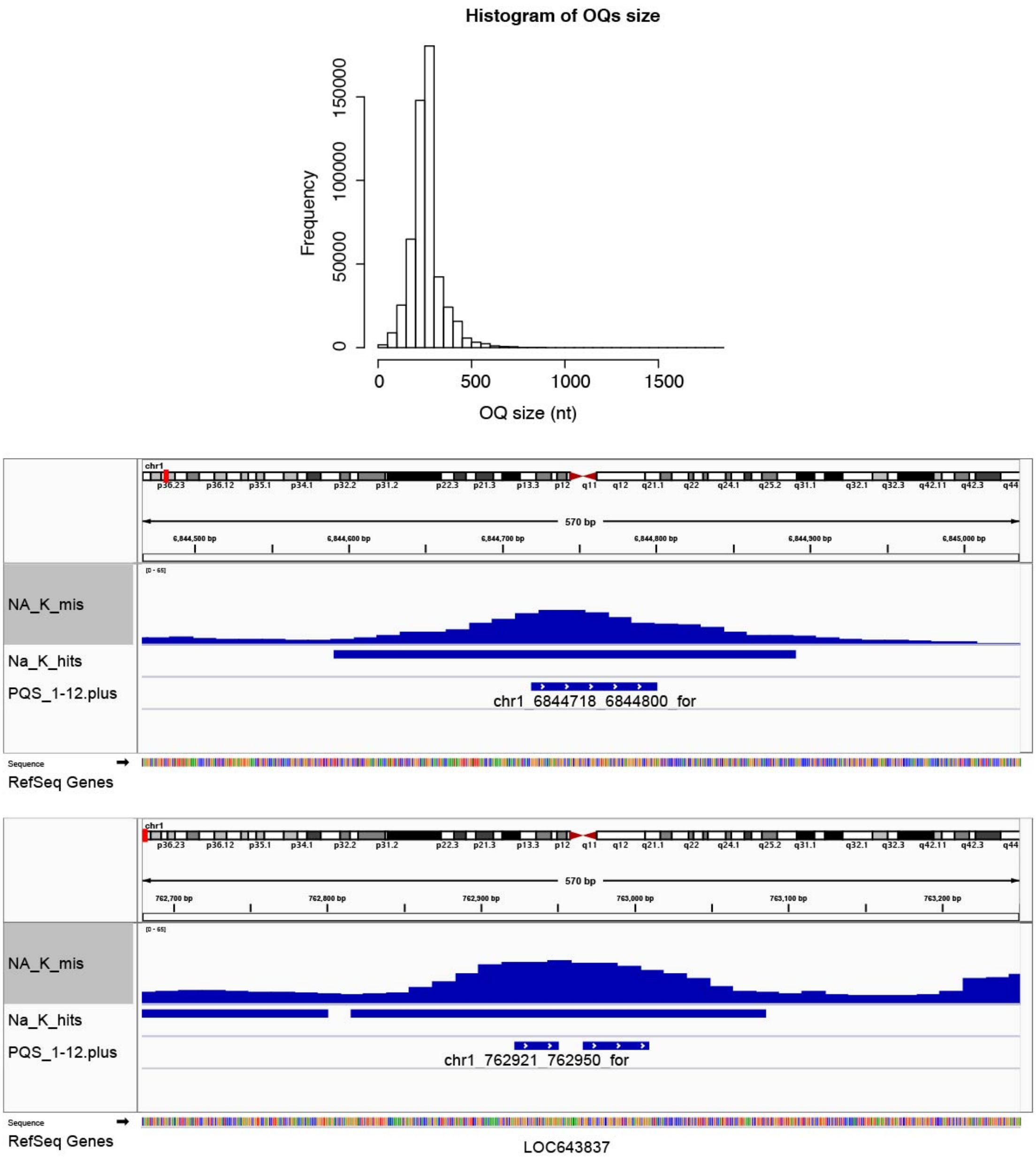

Supplementary Figure S13: OQs region width in G4-seq. A) Histogram of OQs size distribution in G4-seq, K^+ condition. **B)** Two different examples of mismatch tracks on the forward strand (blue peaks "Na_K_plus") at regions with one PQ (above) or two adjacent ones (below), to show the limited resolution problem. "Na_K_hits" intervals show OQs in K^+, forward strand; "PQS_1-12_plus" intervals represent PQS of the form $G_{3+}L_{1-12}$.

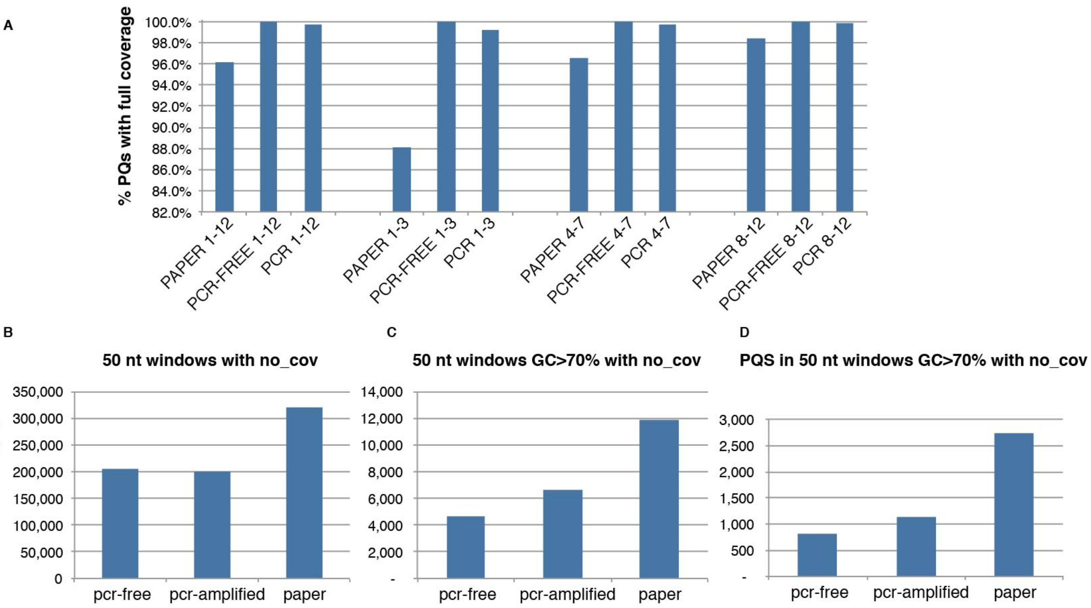

Supplementary Figure S14: Coverage comparison for the improved G4-seq method. A) Percentage of PQS with full coverage for the Human species in the published G4-seq method (label PAPER), in the PCR-FREE method (label PCR-FREE) and in a variant with Li^+ but with PCR amplification (label PCR) for PQS motifs with different loop length: all loops of length 1 to 12, 1 to 3, 4 to 7 and 8 to 12 nucleotides, from left to right, respectively. **B)** Number of 50 nt windows from chromosome 1 for the three different method listed in A) with no coverage (no_cov). **C)** Number of 50 nt windows from chromosome 1 with GC content above 70% with no coverage for the same categories as in A). **D)** Number of 50 nt windows from chromosome 1 with GC content above 70% and with a PQS motif ($G_{3+}L_{1-12}$) having no coverage in the three methods listed in A).

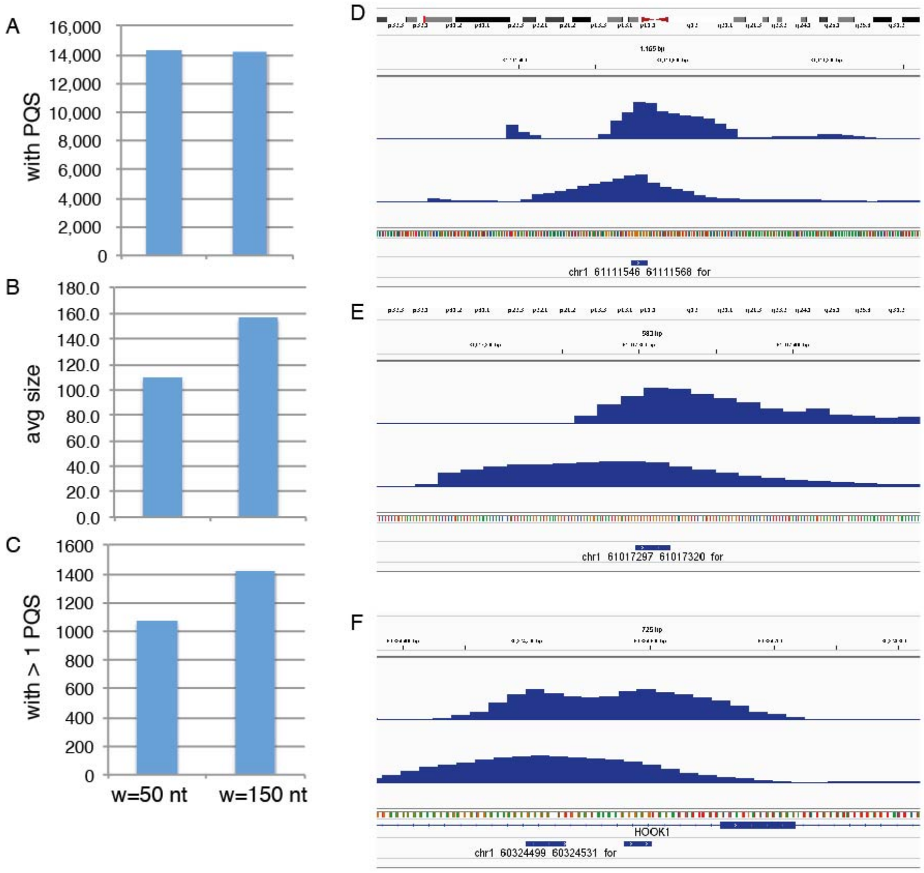

Supplementary Figure S15: Improved OQ resolution for the improved G4-seq method. A) Number of OQs containing a PQS motif ($G_{3+}L_{1-7}$) in chromosome 1 of the Human sample: comparison of the analysis performed with an averaging window of 50 or 150 nucleotides. **B)** Average size of OQs in chromosome 1 for the same categories as in A). **C)** Number of OQs containing more than one PQS motif ($G_{3+}L_{1-7}$) in chromosome 1 of the Human sample. Higher window size leads to worst resolution and therefore the aggregation of G4 motifs in close proximity within the same OQ. **D)** and **E)** Examples showing the improved peak resolution (blue coverage tracks) at PQS motifs (blue interval tracks below) for the 50 nt analysis (top track) compared to 150 nt (bottom track). **F)** Example showing that the analysis with 50 nt is able to distinguish PQS motifs in close proximity. Legend as in D) and E).

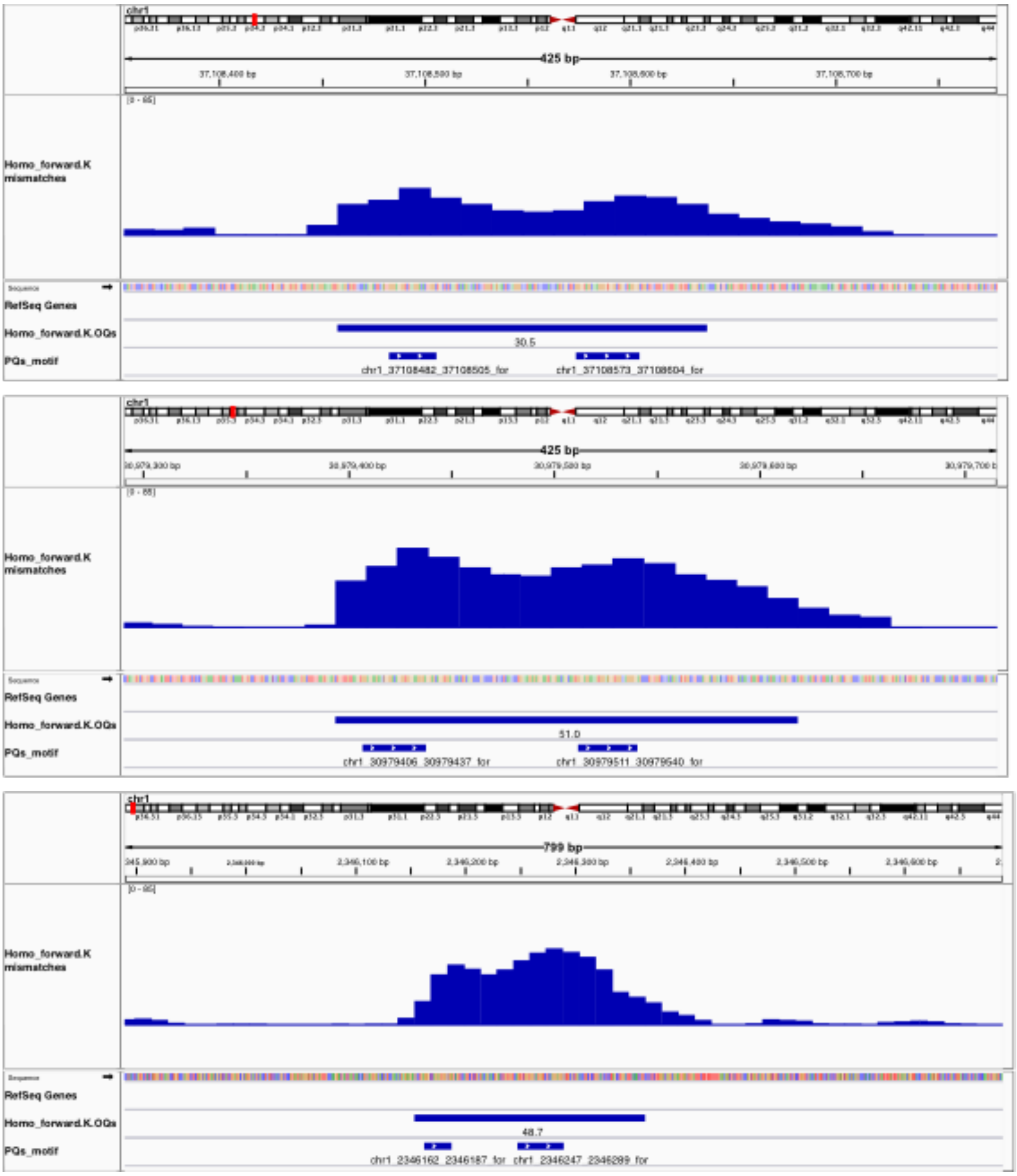

Supplementary Figure S16: Mismatch percentage profiles for 3 selected examples of PQS motifs in close proximity. Three different examples of mismatch tracks on the forward strand in the K^+ vs Li^+ condition (blue peaks) at regions with two adjacent PQS ($G_{3+}L_{1-7}$) (blue intervals below, "PQs_motif") less than 100 nucleotides apart., Two different peaks are visible within, suggesting the formation of different G-quadruplex structures identified as separate with the improved method.

Supplementary Table S1: Full scientific for all the species analysed and short names used as convention in text and figures.

Scientific name	Short name
Homo sapiens	human
Mus Musculus	mouse
Danio rerio	zebrafish
Drosophila melanogaster	Drosophila
Caenorhabditis elegans	C. elegans
Saccharomyces cerevisiae	Saccharomyces
Leishmania major	Leishmania
Trypanosoma brucei	Trypanosoma
Plasmodium falciparum	Plasmodium
Arabidopsis thaliana	Arabidopsis
Escherichia coli	E. coli
Rhodobacter sphaeroides	Rhodobacter

Supplementary Table S2: PQS of various categories and their prevalence in the respective genome. Motif legend (regular expression syntax): $G_{3+}L_{1\text{-}7}$ = $(G_{3+}N_{1\text{-}7})\{3,\}G_{3+}$; $G_{3+}L_{1\text{-}12}$ = $(G_{3+}N_{1\text{-}12})\{3,\}G_{3+}$; $G_{2+}L_{1\text{-}12}$ = $(G_{2+}N_{1\text{-}12})\{3,\}G_{2+}$.

Species (Short Name)	# PQS $G_{3+}L_{1\text{-}7}$	# PQS $G_{3+}L_{1\text{-}12}$	# PQS $G_{2+}L_{1\text{-}12}$
Arabidopsis	1,232	2,849	254,557
C. elegans	2,226	4,291	211,825
zebrafish	50,974	100,655	3,076,040
Drosophila	10,182	22,511	562,924
E. coli	52	131	28,289
human	361,424	705,580	13,948,893
Leishmania	11,462	16,988	295,841
mouse	491,854	786,458	11,770,350
Plasmodium	306	193	7,211
Rhodobacter	633	1,990	71,234
Saccharomyces	38	143	27,728
Trypanosoma	1,646	3,231	168,070

Supplementary Table S3: OQs structural categories in K^++PDS condition. Counts for the same motifs shown in Figure 3A and fold enrichments for all species, calculated as explained in Figure 3E (see Methods).

Genome	# all OQs	# $G_{3+}L_{1\text{-}7}$	# $G_{3+}L_{8\text{-}12}$	# $G_2L_{1\text{-}12}$	# Other	Fold enrich. $G_{3+}L_{1\text{-}7}$	Fold enrich. $G_{3+}L_{8\text{-}12}$	Fold enrich. $G_2L_{1\text{-}12}$	Fold enrich. Other
human	1,376,425	299,879	210,429	771,716	94,401	19.9	13.3	1.7	0.1
mouse	1,746,863	416,178	203,409	892,250	235,026	18.9	11.8	1.8	0.2
zebrafish	321,230	36,031	27,177	175,892	82,130	50.8	38.7	3.9	0.3
Drosophila	55,263	9,174	8,244	32,425	5,420	26.5	19.2	2.0	0.1
C. elegans	10,776	1,921	956	6,462	1,437	83.5	48.6	3.8	0.2
Saccharomyces	502	35	39	314	114	52.5	117.0	3.9	0.3

Leishmania	36,941	10,153	3,860	21,490	1,438	8.9	6.2	1.0	0.1
Trypanosoma	10,666	993	774	7,467	1,432	22.4	13.9	1.9	0.2
Plasmodium	326	90	16	120	100	272.7	48.5	27.7	0.3
Arabidopsis	11,953	780	736	7,552	2,885	93.6	84.9	4.1	0.3
E. coli	560	45	46	419	50	136.4	34.6	1.7	0.2
Rhodobacter	2,291	434	479	1,361	17	14.3	7.7	0.7	0.1

Supplementary Table S4: Genome and annotation files sources for each species.

Species	Genome source	Annotation source
Homo sapiens	http://hgdownload.cse.ucsc.edu/downloads.html (*hg19*)	http://emea.support.illumina.com/sequencing/sequencing_software/igenome.html; https://www.gencodegenes.org/releases/19.html
Mus musculus	http://hgdownload.cse.ucsc.edu/downloads.html (*mm10*)	ftp://ftp.ensembl.org/pub/release-87/gff3/mus_musculus/
Danio rerio	http://hgdownload.cse.ucsc.edu/downloads.html (*danRer10*)	ftp://ftp.ensembl.org/pub/release-87/gff3/danio_rerio/
Drosophila melanogaster	http://hgdownload.cse.ucsc.edu/downloads.html (*dm6*)	ftp://ftp.ensembl.org/pub/release-87/gff3/drosophila_melanogaster/
Caenorhabditis elegans	ftp://ftp.wormbase.org/pub/wormbase/releases/WS220/species/c_elegans/	ftp://ftp.wormbase.org/pub/wormbase/releases/WS220/species/c_elegans/
Saccharomyces cerevisiae	http://hgdownload.cse.ucsc.edu/downloads.html (*sacCer3*)	ftp://ftp.ensembl.org/pub/release-87/gff3/saccharomyces_cerevisiae/
Leishmania major	ftp://ftp.sanger.ac.uk/pub/project/pathogens/Leishmania/major/Archive/LmjF_v6.1_20131105/fasta/ (*ce11*)	ftp://ftp.sanger.ac.uk/pub/project/pathogens/gff3/CURRENT
Trypanosoma brucei	ftp://ftp.sanger.ac.uk/pub/project/pathogens/Trypanosoma/brucei/Tb927/	ftp://ftp.sanger.ac.uk/pub/project/pathogens/gff3/CURRENT
Plasmodium falciparum	http://plasmodb.org/common/downloads/release-28/Plasmodium/fasta/	http://plasmodb.org/common/downloads/release-28/Pfalciparum3D7/gff/data/
Arabidopsis thaliana	ftp://ftp.ensemblgenomes.org/pub/plants/release-31/fasta/arabidopsis_thaliana/dna/	https://www.arabidopsis.org/download/index-auto.jsp?dir=%2Fdownload_files%2FGenes%2FTAIR10_genome_release%2FTAIR10_gff3
E. coli	ftp://ftp.ensemblgenomes.org/pub/bacteria/release-34/	ftp://ftp.ensemblgenomes.org/pub/bacteria/release-34/gff3/bacteria_0_collection/escherichia_coli_str_k_12_substr_mg1655/
Rhodobacter sphaeroides	ftp://ftp.ensemblgenomes.org/pub/bacteria/release-30/	ftp://ftp.ensemblgenomes.org/pub/bacteria/release-30/gff3/bacteria_0_collection/rhodobacter_sphaeroides_2_4_1/

Supplementary References

54. Rodriguez, R., Müller, S., Yeoman, J.A., Trentesaux, C., Riou, J.F., Balasubramanian, S. (2008) A novel small molecule that alters shelterin integrity and triggers a DNA-damage response at telomeres. J. Am. Chem. Soc., 130, 15758-15759.

55. Aird, D., Ross, M.G., Chen, W.S., Danielsson, M., Fennell, T., Russ, C., Jaffe, D.B., Nusbaum, C., Gnirke, A. (2011) Analyzing and minimizing PCR amplification bias in Illumina sequencing libraries. Genome Biol. 12, R18.

56. Lim, K.W., Ng, V.C., Martín-Pintado, N., Heddi, B., Phan, A.T. (2013) Structure of the human telomere in Na+ solution: an antiparallel (2+2) G-quadruplex scaffold reveals additional diversity. Nucleic Acids Res., 41,10556-10562.

57. Tucker, B.A., Hudson, J.S., Ding, L., Lewis, E., Sheardy, R.D., Kharlampieva, E., Graves, D. (2018) Stability of the Na+ Form of the Human Telomeric G-Quadruplex: Role of Adenines in Stabilizing G-Quadruplex Structure. A.C.S. Omega, 3, 844-855

58. Kozarewa, I., Ning, Z., Quail, M.A., Sanders, M.J., Berriman, M., Turner, D.J. (2009) Amplification-free Illumina sequencing-library preparation facilitates improved mapping and assembly of (G+C)-biased genomes. Nat. Methods, 6, 291-5.
